# Systematic validation and assessment of immunohistochemical markers for central nervous system pathology in cetaceans, with emphasis on auditory pathways

**DOI:** 10.1371/journal.pone.0269090

**Published:** 2022-06-01

**Authors:** Ksenia Orekhova, Cinzia Centelleghe, Giovanni Di Guardo, Jean-Marie Graïc, Bruno Cozzi, Davide Trez, Ranieri Verin, Sandro Mazzariol

**Affiliations:** 1 Department of Comparative Biomedicine and Food Science, University of Padova, Legnaro, Padova, Italy; 2 Faculty of Veterinary Medicine, University of Teramo, Località Piano d’Accio, Teramo, Italy; Animal Health Centre, CANADA

## Abstract

Cetacean neuropathology is a developing field that aims to assess structural and neurochemical changes involved in neurodegenerative, infectious and traumatic processes, however markers used previously in cetaceans have rarely undergone systematic validation. This is a prerequisite to investigating the potential damage inflicted on the cetacean auditory system by anthropogenic noise. In order to assess apoptotic, neuroinflammatory and structural aberrations on a protein level, the baseline expression of biomarker proteins has to be characterized, implementing a systematic approach to validate the use of anti-human and anti-laboratory animal antibodies in dolphin tissues. This approach was taken to study 12 different antibodies associated with hypoxic-ischemic, inflammatory, plastic and excitatory-inhibitory changes implicated in acoustic trauma within the ventral cochlear nuclei and inferior colliculi of 20 bottlenose dolphins (*Tursiops truncatus*). Out of the 12 tested antibodies, pro-apoptotic protease factor 1 (Apaf-1), diacylglycerolkinase-ζ (DGK-ζ), B-cell lymphoma related protein 2 (Bcl-2), amyloid-β peptide (Aβ) and neurofilament 200 (NF200) were validated employing Western blot analyses and immunohistochemistry (IHC). The results of the validation process indicate specific patterns of immunoreactivity that are comparable to those reported in other mammals, thus suggesting a key panel of IHC biomarkers of pathological processes in the cetacean brain. As a consequence, the antibodies tested in this study may constitute a valid tool for supporting existing diagnostic methods in neurological diseases. The approach of systematic validation of IHC markers in cetaceans is proposed as a standard practice, in order for results to be transparent, reliable and comparable.

## Introduction

The pathogenesis of the most common pathological changes of the central nervous system (CNS) of whales and dolphins lacks systematic characterization. Detailed and complete studies on protein dynamics describing changes in protein properties as drivers and responses to pathophysiological mechanisms, including changes in subcellular location (e.g. shift from nucleus to cytoplasm), depletion and up- or down-regulation, are often missing. A better understanding of brain pathology holds potential to investigate the causative role of natural threats, such as infectious agents [1] and algal biotoxins [2], but also anthropogenic disturbances including noise [3] and various environmental contaminants [4], particularly after mass stranding events.

The brain is extracted during routine necropsy procedures [5] and sampling from six to eight well defined areas for subsequent microscopic examination in all well-conserved carcasses is strongly recommended [6]. In addition to the above mentioned protocol, the examination of two to three central auditory pathway components using IHC, as proposed for laboratory and terrestrial species [7], could facilitate comparisons between both individuals and species, and visualizing pathological processes before the appearance of morphological lesions could reveal significant changes associated with auditory and non-auditory pathology.

The most common pathological conditions of the CNS often do not produce relevant grossly visible changes, and histopathological examination could reveal different forms of damage including apoptosis [8, 9], inflammation associated to relevant pathogens [1, 10], and neoplasia [11]. These and other changes such as vascular pathology related to free radical accumulation, myelinopathy and synaptic remodeling [12–14] are not pathognomonic of a single cause but they are differentially associated with some of them. Examples include inflammation marked by encephalitis and vasculitis triggered by infections (e.g. DMV [10]), blast or acoustic trauma [15, 16] and neurodegeneration involving deposits of misfolded proteins resulting from biotoxin accumulation (e.g. β-N-methylamino-L-alanine) [4]. By providing stronger evidence for a specific source of damage and anticipating morphological evaluation, IHC allows for a deeper insight into pathological mechanisms and represents considerable support in the microscopic examination of the CNS. IHC markers have previously been assessed in cetacean brains for both homeostatic mechanisms (e.g. calcium-binding proteins [17–19]), as well as neurodegenerative changes, e.g. Aβ [20–23], NFT, TDP-43 [4] and caspases [24]. However, systematic validation involving at least two orthogonal methods and the consistent use of positive control tissues is found in relatively few of those studies [4, 19].

This is particularly relevant for markers of noise exposure, since the auditory sense predominates cetacean perception [25]. In fact, while methods to investigate harmful effects of underwater acoustic sources already exist for the inner ear of cetaceans [3, 26], they are limited by carcass decomposition (i.e. they can be applied only a few hours from the death of the animals) and they may not provide conclusive information on other changes, such as tinnitus and non-auditory CNS pathology.

In the biomedical literature on humans and laboratory animals, numerous selected biomarkers reflect the above-mentioned mechanisms, and have previously proven useful in the assessment of acute to chronic noise-induced pathology in laboratory animals. Inducible nitric oxide synthase (iNOS) and malondialdehyde (MDA) [27] indicate peracute, free radical build-up in perivascular tissues inducing vasoconstriction and ischemia/reperfusion damage [12]. Glutamate decarboxylase^67^ (GAD^67^) may mark the disequilibrium of excitatory/inhibitory transmission due to build-up of excitotoxic glutamate [28]. Extracellular Aβ-accumulation [29] may exacerbate dysregulation of vascular tone via both vasoconstriction [30] and vasodilation [31], and induce neuroinflammation through interactions with tumor necrosis factor α (TNFα) [16]. Apaf-1 [8], DGK-ζ [9] and Bcl-2 [7] are all involved in programmed cell death as apoptotic and anti-apoptotic signal molecules. NF200 is an integral cytoskeletal protein, and may undergo proteolysis as a consequence of neurodegeneration [32] and focal ischemia [33].

This cycle of deterioration leads to further cell death on one hand [29], but may also contribute to limited tissue repair by reducing the resulting cellular debris. This may enable the formation of new synaptic connections [13], or neuroplasticity, where synaptic sprouting induces an increased expression of growth associated protein 43 (GAP43) [34] and focally decreases synaptophysin expression [27]. Quaking protein-7 (QKI-7) is one of the proteins upregulated during developmental myelination that may be reactivated in the course of a myelinopathy as a form of attempted repair [14]. The effects of these and further changes may compromise tissue function for up to years after their occurrence [35], hence their detection could characterize pathological processes in dolphins, including acoustic trauma.

In order to implement existing protocols [5, 23] by increasing the numbers of systematically validated antibodies, in the present study we tested two crucial central auditory processing centers—the ventral cochlear nuclei (VCN) and inferior colliculi (IC)—of 20 bottlenose dolphins (*Tursiops truncatus*). Thereby, we could integrate the requirements of representative brain sampling with the possibility to detect auditory pathology using structures that are easy to identify and sample. The timing, severity and distribution of the lesions, combined with an investigation of a key panel of biomarkers in the central auditory pathways, may help derive the cause of a pathology that cannot otherwise be ascertained.

The aim of this study is to establish a baseline for comparison in the characterization of cetacean CNS pathology. This could help create an IHC “fingerprint” of specific diseases and, eventually corroborate morphopathological findings associated with noise-related damage.

## Materials and methods

### Animals and tissue processing

Brain tissues were sampled from 20 bottlenose dolphins archived in the Mediterranean Marine Mammals Tissue Bank of the University of Padova. The brains originated from both stranded and captive cetaceans with a decomposition and conservation code (DCC) of 1 and 2, according to the available guidelines for cetacean *post mortem* investigation [5]. Table 1 summarizes pertinent information on the investigated dolphins, with additional information on their sex, body length, full histopathological findings of the CNS, and the most probable cause of death provided in the supplementary materials. Brains had been extracted within 24 hours after death, cut into 1 cm-thick coronal sections, fixed in 10% neutral-buffered formalin and washed in phosphate buffer (0.1 M, pH: 7.4) prior to paraffin embedding [36, 37]. When available, the right VCN (n = 11) and IC (n = 17) of each animal were sampled according to the protocol in Supplementary Materials, and routinely processed for histological and IHC analyses as previously reported [38]. In the case that the selected brain nuclei were not intact on the right side (ID114 VCN, ID142 VCN, ID145 VCN and IC, ID192 IC, ID196 VCN, ID319 VCN), the left side was sampled. In few animals, some nuclei were no longer available bilaterally (VCN of ID20, ID133, ID139 and ID159; IC of ID343) and could not be included in this study.

**Table 1:** Summary of the ID, age group, predominant pathological process detected histologically the CNS, and pooled histoscore averages of the protein marker immunoreactivity for the VCN and IC of the dolphins of this study (0—No immunoreactivity; 3—Very intense immunoreactivity).

ID	Age/ age group	Categories			Predominant pathological proccess	Histoscore averages for the VCN and IC				
		A	B	C		Apaf-1	DGK-ζ	Bcl-2	Aβ	NF200
**146**	Adult	N	N	Y	Cell death and degeneration	1.42^**nc**^; 1.71^**ac**^	2.63^**nn**^l; 0^**nc**^	0^**all**^	1.5^**nn**^; 2.15^**nc**^	2.69^**nc**^
**20**	30 y	N	N	O	Cell death and degeneration	0.43^**nc**^; 0.88^**ac**^	1.01^**nnl**^; 0.7^**nc**^	1.09^**n**^c; 0.6^**ac**^	0.9^**nn**^; 1.07^**nc**^; 2.0^**ac**^	2.39^**nc**^
**89**	5 y	N	N	Y	Cell death and degeneration	0.7^**nc**^; 1.57^**ac**^	2.16^**nnl**^; 0.47^**nc**^	0.6^**nc**^; 0.55^**ac**^	2.4^**nn**^; 1.08^**nc**^; 1.55^**ac**^	2.69^**nc**^
**139**	30 y	N	N	O	Cell death and degeneration	0.65^**nc**^; 0.9^**ac**^	1.45^**nnl**^; 0.68^**nc**^	0^**all**^	0.93^**nn**^; 0.8^**nc**^; 2.1^**ac**^	2.98^**nc**^
**159**	40 y	N	N	O	Inflammation; Cell death and degeneration	1.0^**nc**^; 1.46^**ac**^	0.3^**nnl**^; 0.84^**nc**^	0^**all**^	0.6^**nn**^; 1.0^**nc**^, 2.0^**ac**^	2.94^**nc**^
**319**	Old	N	N	O	Inflammation; Hypoxia	1.45^**nc**^; 2.13^**ac**^	0.15^**nnl**^; 0.72^**nc**^	0.1^**nc**^; 0.59^**ac**^	1.92^**nn**^; 1.22^**nc**^; 1.7^**ac**^	2.61^**nc**^
**344**	Sub-adult	N	P	Y	Hemodynamic disorder	0.95^**nc**^; 1.76^**ac**^	0.99^**nnl**^; 0.74^**nc**^	0^**all**^	2.17^**nn**^; 1.0^**nc**^; 1.9^**ac**^	2.78^**nc**^
**192**	Adult	N	N	Y	None detected	0.8^**nc**^; 1.64^**ac**^	1.79^**nnl**^; 0.52^**nc**^	0^**nc**^; 0.75^**ac**^	1.63^**nn**^; 1.48^**nc**^; 1.9^**ac**^	2.69^**nc**^
**196**	Old	P	P	O	Inflammation (associated with T. gondii)	0.92^**nc**^; 1.75^**ac**^	1.5^**nnl**^; 0.62^**nc**^	0.6^**nc**^; 0.6^**ac**^	1.39^**nn**^; 1.48^**nc**^; 1.85^**ac**^	2.85^**nc**^
**95**	Adult	P	P	O	Hemodynamic disorder; Inflammation	0.87^**nc**^; 1.98^**ac**^	1.3^**nnl**^; 0.89^**nc**^	1.0^**nc**^; 0.4^**ac**^	1.9^**nn**^;1.23^**nc**^; 2.05^**ac**^	2.59^**nc**^
**107**	9 y	P	N	Y	Inflammation; Cell death	1.04^**nc**^; 1.89^**ac**^	2.38^**nnl**^; 0.41^**nc**^	0^**all**^	1.78^**nn**^; 1.4^**nc**^; 1.85^**ac**^	2.7^**nc**^
**133**	>30 y	P	P	O	Inflammation; Cell death	0.8^**nc**^; 1.79^**ac**^	1.72^**nnl**^; 0.9^**nc**^	0^**all**^	1.77^**nn**^; 1.13^**nc**^; 2.0^**ac**^	2.91^**nc**^
**142**	Old	P	P	O	Inflammation (associated with T. gondii)	0.6^**nc**^; 1.32^**ac**^	0.48^**nnl**^; 0.27^**nc**^	0^**all**^	0.8^**nn**^; 0.67^**nc**^; 2.0^**ac**^	2.47^**nc**^
**165**	Old	P	P	O	Inflammation (associated with T. gondii)	0.37^**nc**^; 1.99^**ac**^	0.34^**nnl**^; 0.94^**nc**^	0^**all**^	2.05^**nn**^; 1.12^**nc**^; 1.8^**ac**^	2.81^**nc**^
**201**	Old	P	P	O	Inflammation (associated with DMV)	0.86^**nc**^; 2.05^**ac**^	0.9^**nnl**^; 1.01^**nc**^	0^**all**^	1.77^**nn**^; 1.1^**nc**^; 2.02^**ac**^	2.96^**nc**^
**203**	Old	N	P	O	Cell death and degeneration; Hypoxia	1.11^**nc**^; 1.03^**ac**^	0.05^**nnl**^; 1.21^**nc**^	0^**all**^	0.0^**nn**^; 1.38^**nc**^; 1.75^**ac**^	1.78^**nc**^
**114**	9 d	C	C	C	Cell death and degeneration	1.59^**nc**^; 1.88^**ac**^	2.07^**nnl**^; 0.72^**nc**^	1.09^**nc**^; 0.82^**ac**^	2.34^**nn**^; 1.3^**nc**^; 1.45^**ac**^	2.27^**nc**^
**144**	9 d	C	C	C	Cell death	1.8^**nc**^; 1.84^**ac**^	1.53^**nnl**^; 0.99^**nc**^	0.95^**nc**^; 0.2^**ac**^	2.76^**nn**^; 1.32^**nc**^; 1.65^**ac**^	2.71^**nc**^
**145**	9 d	C	C	C	Cell death	1.54^**nc**^; 1.73^**ac**^	2.62^**nnl**^; 0.02^**nc**^	1.17^**nc**^; 0.42^**ac**^	2.7^**nn**^; 1.31^**nc**^; 1.6^**ac**^	2.79^**nc**^
**343**	Calf	C	C	C	Cell death	1.79^**nc**^; 1.4^**ac**^	0.97^**nnl**^; 1.05^**nc**^	0^**all**^	2.78^**nn**^; 1.0^**nc**^; 1.4^**ac**^	2.92^**nc**^

Columns A, B and C apply to the grouping of the animals according to microscopic changes in hematoxylin-eosin (HE)-stained slides (A), independent IHC slide analysis (B), and age (C). N—adults without visible lesions in HE-slides or observed aberration in the IHC slides; P—adults with observed microscopic lesions/aberrant IHC-patterns, Y—young (<30 y.o.) adult, O—old (≥30 y.o.) adult. C—cal. M—male; F—female. Subcellular compartments: ac—astrocyte cytoplasm; all—all cell types and compartments; an—astrocyte nuclei; nc—neuronal cytoplasm; nn—neuronal nuclei; nnl—neuronal nucleoli.

Four μm-sections were cut and mounted onto TOMO^®^ Adhesion Microscope Slides (Matsunami Glass). A semi-automatic HE-staining using a Leica Autostainer XL (Leica Biosystems Nussloch GmbH) was performed and the slides were then coverslipped using a mixture of Eukitt^®^ (ORSAtec GmbH) and xylene, and checked for CNS lesions by a blinded board-certified pathologist using a light microscope. An overview of the significant histopathological findings is visualized in the supplementary files.

### Antibody validation process

Systematic validation of the selected antibodies for bottlenose dolphin species followed published approaches [39, 40]. Besides testing the IHC immunoreactivity (IR) on bottlenose dolphin tissues, selecting adequate positive and negative control tissues and including sections not treated with the primary antibody (blank sections), orthogonal Western blot (WB) or BLAST analyses were performed to confirm the presence and specificity of the antigen. Antibodies were then selected when a clear IR was detected by IHC and when the antigen specificity was confirmed in the examined tissues by at least one of the two orthogonal techniques.

### Western blot analysis

Protein extraction for WB analysis was performed as reported by De Vreese and colleagues (2019) [38], using frozen brain tissue from bottlenose dolphin, fin whale, Cuvier’s beaked whale, striped dolphin and sperm whale specimens. Overnight incubation at 4°C followed, using polyclonal rabbit Apaf-1 (Enzo, #ADI-905-179-100, 1:1000), anti-DGK-ζ (MyBiosourse, #MBS2026991, 1:500), anti-Bcl-2 (Abcam, #ab196495, 1:1000), anti-MDA (Abcam, #ab6463, 1:1000) and anti-iNOS (Abcam, #ab15323, 1:250); a monoclonal recombinant rabbit anti-Aβ (ThermoFisher Scientific, #700254, 1:500); and monoclonal mouse anti-NF200 (Sigma-Aldrich, #N0142), anti-GAD^67^ (Sigma-Aldrich, #MAB5406, 1:2000), anti-GAP43 (Sigma-Aldrich, #MAB347, 1:1000), anti-TNFα (Santa Cruz, #sc-52746, 1:200), anti-QKI-7 (Antibodies-online, #ABIN1304925, 1:1000) and anti-synaptophysin (Dako; #M7315, 1:1000). After several washes in TBS-T, the membrane was incubated with an anti-rabbit peroxidase-conjugated secondary antibody (ThermoFisher Scientific, #32260) for 1 hour at room temperature. In order to visualize immunoreactive bands, a chemiluminescent detection kit (SuperSignal West Pico Chemiluminescent Substrate, ThermoFisher Scientific) and the iBright machine (ThermoFisher Scientific) were employed.

### BLAST analysis

In the case of inconclusive WB results, target proteins of employed antibodies were additionally tested against the predicted *T*. *truncatus* proteome made available by the NCBI (https://blast.ncbi.nlm.nih.gov/Blast.cgi?PAGE_TYPE=BlastSearch&PROG_DEFAULTS=on&PAGE=Proteins&PROGRAM=blastp&BLAST_SPEC=OGP__9739__20365&DATABASE=RefseqProteins/Refseq_Protein_9739). The NCBI amino acid sequences were selected according to their similarity to the proteins targeted by the antibodies utilized in this study. Dolphin protein isoforms with the closest molecular weight and sequence homology to the requested sequence were ranked in terms of degree of identity (in percent) and E-Value.

### IHC analysis

In order to validate the use of the primary antibodies for bottlenose dolphin, validated positive control tissues were first selected for each antibody. IHC protocols were adjusted using a Ventana Benchmark^®^ GX semi-automatic immunostainer containing a kit of automatically dispensed reagents including the secondary antibody and HRP-conjugated polymer (UltraView Universal DAB; Ventana Medical Systems).

Primary antibodies included the same ones tested in the WB procedures, with dilutions optimized for IHC: polyclonal rabbit anti-Apaf-1 (dilution: 1:500), anti-DGK-ζ (1:100), anti-Bcl-2 (1:150), anti-MDA (1:100) and anti-iNOS (1:100); monoclonal recombinant rabbit anti-Aβ (1:1000); monoclonal mouse anti-NF200 (1:400), anti-GAD^67^ (1:500), anti-GAP43 (1:1000), anti-TNFα (1:50), anti-QKI-7 (1:100) and anti-synaptophysin (1:50).

In each IHC run, a positive control, as well as blank sections of the same tissues were included to exclude unspecific binding of the secondary antibody and of the HRP-conjugated polymer. Internal negative controls were evaluated in each slide according to the specific cell type reactivity as described by the antibody producer and existing biomedical literature.

Additionally, a polyclonal rabbit anti-human *T*. *gondii* antibody (MyBioSource; # MBS373041; 1:80), was used on the brain tissue of animals in which molecular analyses had confirmed the presence of the protozoa [41] in order to investigate the localization of *T*. *gondii* itself in relation to the IR patterns of selected antibodies in neuroinflammatory foci.

### Animal categorization and semi-quantitative scoring

Adult animals were grouped into pathological (*P*)/non-pathological (*N*) categories according to the presence or absence of microscopic changes (Table 1, column A) including 1) neuronal degeneration and necrosis as marked by cell hypereosinophilia, loss of subcellular detail, darkened or shrunken appearance, and satellitosis; 2) cerebral edema recognizable by spongiotic foci and widened perivascular spaces; 3) (meningo-)encephalitis evidenced by infiltrates of inflammatory cells, perivascular cuffing, astrocytosis and/or presence of parasitic agents, glial nodes and/or gemistocytes. Previously established molecular positivity to *T*. *gondii* (ID142, ID165, ID196) [41] and *Dolphin Morbillivirus* (ID201) [10] were also considered in the classification of the animals, particularly when associated to the aforementioned CNS lesions.

In order to evaluate the potential of IHC to deliver information on the health status of brain tissue, the animals were then categorized independently into adult dolphins with evident CNS pathology/adult dolphins without evident CNS pathology/calf (*P*, *N*, *C*, respectively) categories according to qualitative assessment of only the IHC IR patterns considering the extent, localization and distribution of the IR (Table 1, column B).

Lastly, the animals were again independently grouped into the following categories (Table 1, column C): young adults (≈<30 years old) (Y), old adults (≈≥30 y.o.) (O) and calves (C) to account for age-related changes in expression profiles.

Since the ages of the adult animals were not always known, 5 individuals of known ages (9 days, 3.5 years, 9 years, 30 years, 40 years) were used as references for a scatterplot-based correlation of total length (from tip of the rostrum to the notch of the fluke) to approximate age according to age-length reference ranges for Mediterranean bottlenose dolphin specimens [42] and necropsy findings including tooth wear, body condition, gonad weight and presence of ovarian scars. An age-length estimation curve for the animals with known total body lengths is depicted in S1 Fig, and represents a logarithmic trend line automatically calculated in Microsoft Powerpoint^®^ from a scatterplot of the available length measurements. Adult dolphins with a total length superior to that of ID139 (30 y.o.) were estimated to be ≥ 30 y.o., regardless of their sex.

For each marker, the IR intensity was recorded subjectively for each relevant cell type and subcellular compartment (SC) of each animal and auditory nucleus. A value of *1* (mild), *2* (moderate) or *3* (intense) was assigned on IHC staining by evaluating five high power fields (400x magnification) for each auditory nucleus and animal. In order to assess the relative number of observed SCs of each intensity level, the histoscore (H = (1 * % of intensity score “*1*” SCs) + (2 * % of intensity score “*2*” SCs) + (3 * % of intensity score “*3*” SCs) was calculated. The H-average value for the five fields was then used for the database used for statistical assessment of the various animal groups.

### Statistical analysis

In order to properly evaluate the potential of IHC to deliver information on the health status of brain tissue going beyond routine morphological assessment, three analyses based on the aforementioned categorization of the animals were performed in RStudio^®^. Scores of the semi-quantitative manual scoring for relevant subcellular compartments were tested for normality visually (histograms can be found in S2 Fig) and using the Shapiro-Wilk test. Due to the relatively small sample size, homogeneity of variance was additionally tested using Levene’s test in cases where the Shapiro-Wilks implied normality. Results were considered statistically significant at a *p*-value < 0.05. Animal groups independently categorized into three health categories: adult brains with lesions; adult brains without lesions; and neonate brains according to A) microscopic findings in HE sections B) IHC-patterns and C) age. One-way ANOVAs were used to compare normally distributed marker scores, whereas the Kruskal-Wallis test was implemented on non-normally distributed scores (Bcl-2—neuronal cytoplasm in IC and VCN; DGK-ζ—neuronal cytoplasm, VCN; Apaf-1—astrocyte cytoplasm, VCN; Aβ—neuronal nucleus, VCN; NF200—neuronal cytoskeleton, IC).

The statistically significant results were then compared using the Tukey HSD and Wilcoxon signed-rank test for parametric and non-parametric data, respectively, in order to assess which animal categories displayed relevant differences. Due to the small sample size, three levels of adjustment with increasing restriction for α-error of the Wilcoxon test were used: None, Benjamini-Hochberg and Bonferroni.

## Results

### Antibody validation process

The selected antibodies were developed against human or laboratory animal antigens, so their specificity in cetacean tissues required validation through a well-defined procedure aimed at confirming their IR. WB analyses confirmed reactivity with antigens of adequate molecular weight; alternatively, a basic local alignment search tool (BLAST) allowed to compare protein sequences to sequence databases calculating the statistical significance [39]. Finally, IHC validation in 20 bottlenose dolphins was performed detecting any cross-reactivity of the selected antibodies with unrelated antigens, and cross-reactivity among different species [39]. Antibodies were only selected for further analyses if IHC and at least one of the orthogonal methods (WB/BLAST) confirmed their specificity [40]. Out of the 12 antibodies listed in the methodology, only four encompassed the criteria of contemporary positive IHC reaction and WB (DGK-ζ, Bcl-2, Aβ and NF200). While Apaf-1 was negative in the WB, BLAST analysis and specificity of its IHC-IR in selected positive control tissues, verified its suitability for systematic IHC analysis.

### Western blot analysis

**Fig 1:**
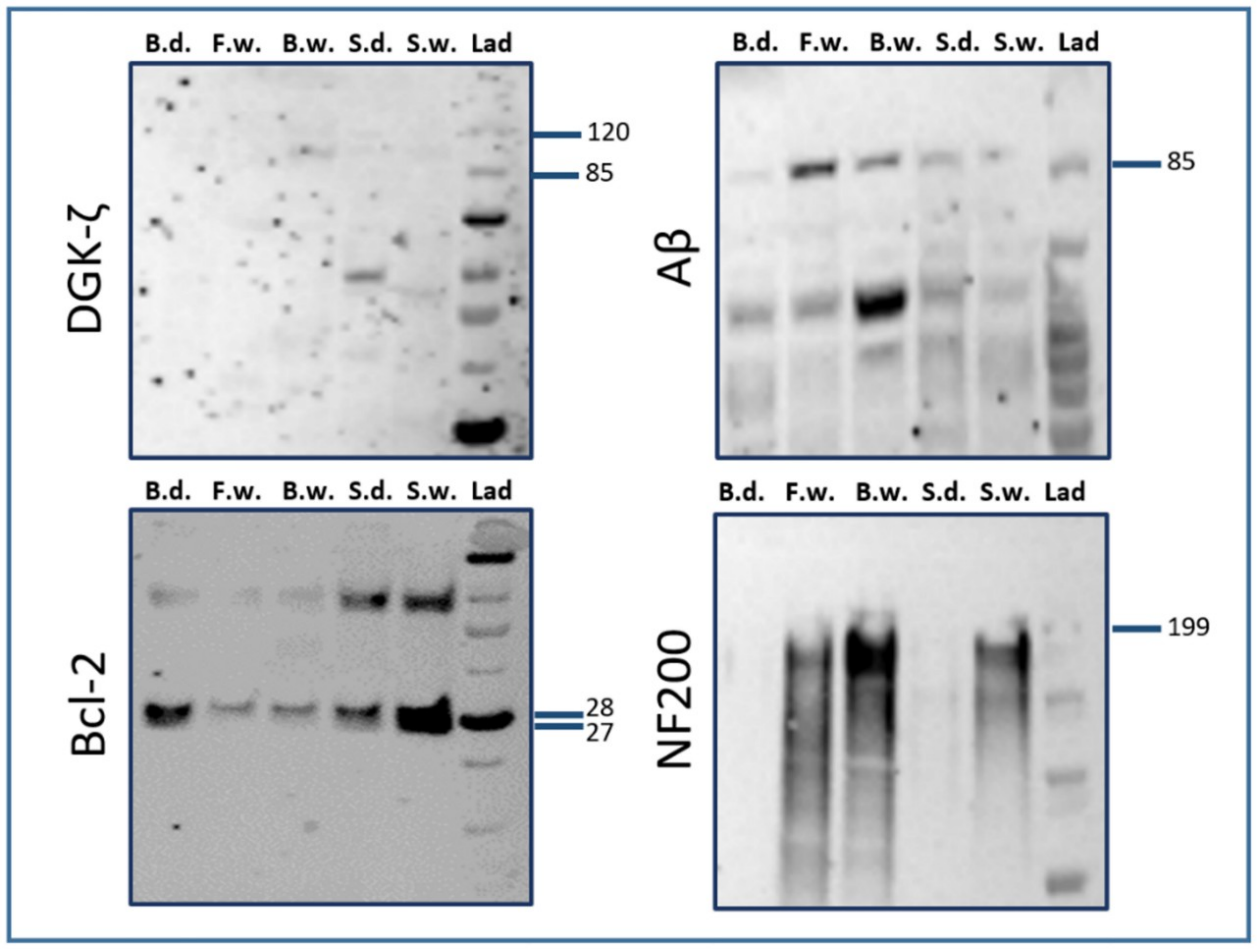
Results of WB analyses for antibodies against DGK-ζ, Bcl-2, Aβ, and NF200. B.d.: Bottlenose dolphin; F.w.: Fin whale; B.w.: Beaked whale; S.d.: Striped dolphin; S.w.: Sperm whale; Lad: Ladder. Labels on the right of each panel signify molecular weight in kDa.

Among all the tested antibodies, WB analysis (see Fig 1) established the specificity of primary antibodies against Bcl-2 (≈26 kDa) and Aβ (≈87 kDa) for brain tissue in bottlenose and striped dolphin (*Stenella coeruleoalba*), as well as for fin whale (*Balaenoptera physalus*), Cuvier’s beaked whale (*Ziphius cavirostris*; henceforth: BW) and sperm whale (*Physeter macrocephalus*). For NF200, IR-bands were detected for fin, beaked and sperm whales at approximately 200 kDa, while for DGK-ζ, a weak signal was observed for BW, sperm whale and striped dolphin at around 100 kDa, which corresponds to the length of human and rat isoforms (≈104 kDa). The antibodies against Apaf-1, MDA iNOS, GAD^67^, GAP43, TNFα, QKI-7 and synaptophysin did not yield detectable results. Unprocessed images of the blots of the successful markers are included in S3 Fig.

### BLAST analysis

WB signal against bottlenose dolphin tissue was not detected for Apaf-1, NF200 and DGK-ζ. However, BLAST analysis against the estimated *T*. *truncatus* proteome showed that proteins with a comparable amino acid sequence are present in this species. Therefore, the IHC patterns were deemed reliable. For DGK-ζ, the homology degree between the mouse (NP_612179.2) and dolphin isoforms X7 and X8 equaled 94.4% (E-value: 0). An 85.83% homology level (E-value: 0) was found between the human Apaf-1-isoform c protein (NP_863651.1) and the dolphin Apaf-1-isoform X5 (XP_019773955.1—the closest protein in terms of molecular weight). For NF200, the homology degree between the mouse (NP_035034.2) and the dolphin neurofilament heavy polypeptide (XP_019805688.2) equaled 79.69% (E-Value: 0).

### IHC analysis

Positive control tissues, antibody concentration, and expected immunoreactive cell types for the antibodies that passed the selection criteria (a clear IHC-pattern in combination with the positivity of at least one of the orthogonal validation methods), as well as reported significance of the cellular pattern are summarized in Table 2.

**Table 2:** Summary of IHC parameters of the herein utilized antibodies, and their roles in the body depending on subcellular location.

Antibody	Dilution	+ CTRL	CNS cell type	Significance of cellular pattern
polyclonal rabbit anti-Apaf-1	1:500	Lung with lymphoma, rat	• Neurons	Cytoplasmic—up-regulated in the process of intrinsic apoptosis (including that in the developing brain);
			• Glia (incl. pericytes)	Nuclear—associated to acute hypoxic conditions;
			• Endothelia	Acellular perivascular leakage—mild hypoxia (likely a common phenomenon in diving mammals) may result in transient, physiological reduction of BBB integrity
polyclonal rabbit anti-DGK-ζ	1:100	Cerebellum, rat	• Neurons	Nuclear—common in morphologically healthy neurons;
			• Glia	Nucleolar—potential interaction with Mdm2 and p53 proteins, which are implicated in nucleolar stress;
				Cytoplasmic—association with pre-apoptotic state following ischemic-hypoxic episodes and auditory insults, may be neuroprotective and can also be associated with neuroplasticity
polyclonal rabbit anti-Bcl-2	1:150	Lymphonode, Canine	• Neurons	Cytoplasmic—low amounts expected in neonatal neurons and as an anti-apoptotic response to cell injury
			• Glia	
monoclonal recombinant rabbit anti-Aβ	1:1000	Neocortex, canine neonate	• Neurons	Cytoplasmic—neurotoxic effect through disruption of calcium homeostasis, organelle and synaptic function;
			• Glia	Nuclear—presumed regulation of apoptosis and potential indicator of neuroprotection against neurodegeneration;
			• Endothelia	Extracellular plaques—presence positively correlated with cognitive decline in Alzheimer’s disease
Monoclonal mouse anti-NF200	1:400	Cerebellum, rat	• Neurons	Cytoskeletal—reduced in cases of traumatic brain injury and hypoxia

+CTRL: Positive control tissue. BBB: Blood-brain barrier.

#### Apaf-1

Cytoplasmic IR was detected in different cell types including astrocytes (ranging in intensity score from *1* to *3*), oligodendrocytes (*3*) and neurons (*1*), the latter showing a stronger immunostaining (*2*) in calves compared to adults. Disseminated glial IR was apparent in all the studied specimens, making a clear differentiation between oligodendrocytes and astrocytes challenging. Apaf-1-IR appeared to be reduced in glial nodules surrounding *Toxoplasma gondii* cysts (Fig 3e).

**Fig 2:**
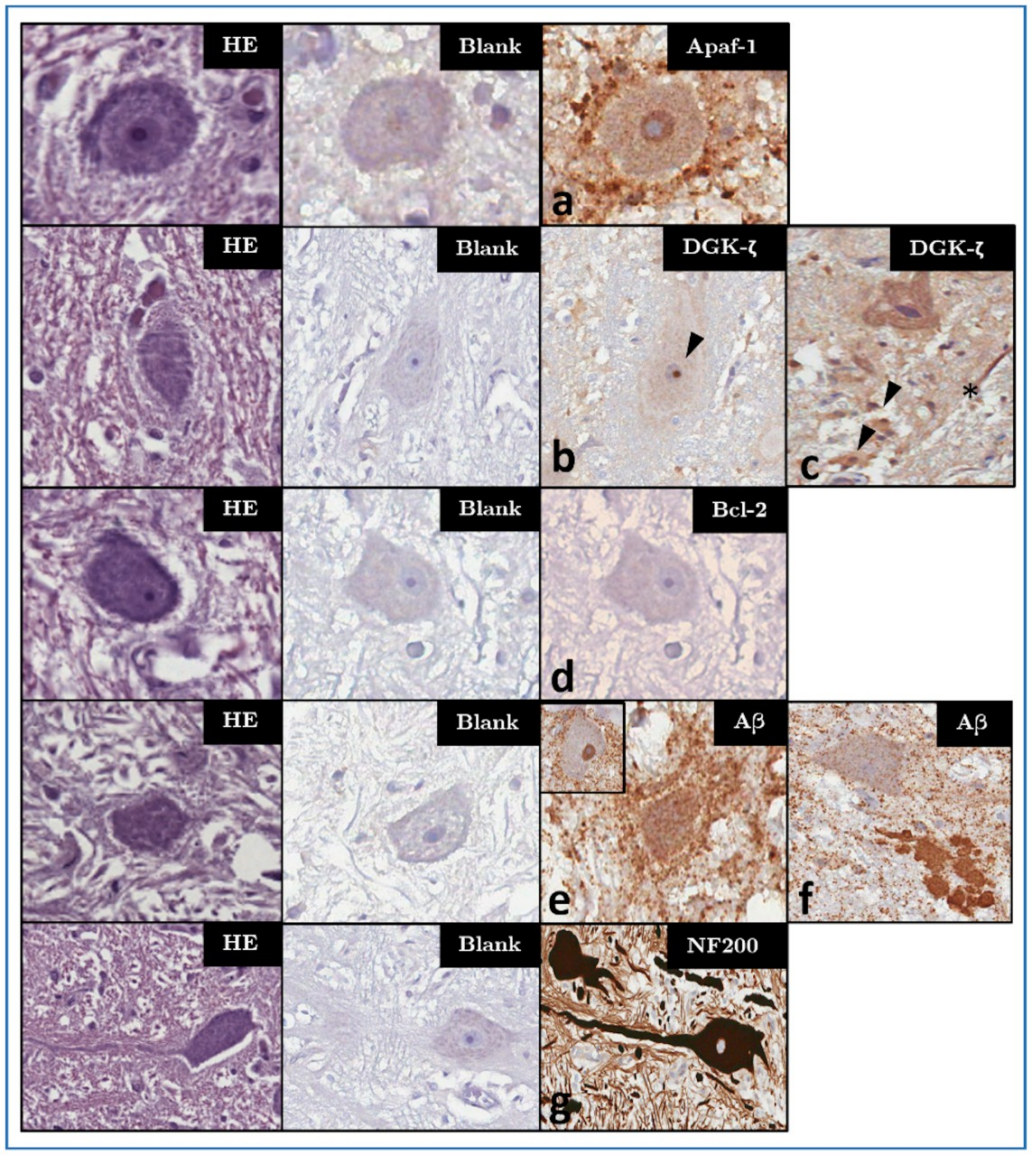
Haematoxylin-eosin/HE (left column), blank control/CTRL (middle column) and IHC patterns (right columns) for the investigated markers—magnification: 400x. Panels marked a-f represent characteristic IHC patterns for each marker: (a) Apaf-1-IR in both neuronal cytoplasm and, unlike in most other examined animals, in the nucleus of ID319. (b) Nucleolar DGK-ζ-IR (arrowhead) in the VCN of a younger adult dolphin without brain lesions (ID146) as compared to (c) its cytoplasmic appearance in the VCN of a bycaught, older adult (ID203). In this animal, multifocal cytoplasmic IR in glial cells (arrowheads), as well as streaks of IR in the neuropil (asterisk) are apparent. (d) Lack of Bcl-2-IR in the VCN of a younger adult dolphin (ID89), representative of most adults. (e) Perineuronal enhancement of glial Aβ-IR in the VCN of a bycaught, hypoxic dolphin (ID203). (f) Aβ-IR marking a coalescing extracellular plaque between the VCN and superior olivary complex of older adult (ID319). An adjacent neuron shows light disseminated granular IR in its cytoplasm, but not in the nucleus, as in the VCN of most investigated adult dolphins <30 y.o. (inset in e). (g) Intense NF200-IR of the dendritosomatic and axonal cytoskeleton of VCN neurons (ID89, representative of all investigated animals).

The VCN neuropil displayed a mild background signal, with a mild to moderate (*1–2*) perisomatic IR surrounding the large neurons of the VCN of three dolphins (ID145, ID319, ID343). VCN neurons of the older dolphins ID319 and ID142—the latter also presenting *T*. *gondii* cysts—additionally displayed a moderate nuclear IR (Fig 2a). In a few large neurons of the VCN, Apaf-1 preferentially localized to the Nissl substance (S4 Fig).

Perivascular IR was prominent in the IC of three of the examined dolphins (ID107, ID201, ID344), variably associated to pericytes (Fig 4c-4d) or to acellular perivascular deposits (S5 Fig). The neuropil also appeared to have a homogeneous, light background.

#### DGK-ζ

DGK-ζ-IR was mainly localized to two different compartments: neuronal nucleoli (intensity: *3*; Fig 2b) and cytoplasm (intensity: *1–2*; Fig 2c). In four adult dolphins and one calf, a clear predominance for the nucleolar pattern emerged in the qualitative assessment. In the others, an overlapping pattern of variable cytoplasmic IR with or without simultaneous nucleolar IR was apparent. In the IC of four adults, and the VCN of 5 adults and one calf, cytoplasmic IR predominated over a weak/undetectable nucleolar staining. No IR was observed in either auditory nucleus of ID142.

Some immunoreactive glial cells were also detected (Fig 2c), at times displaying a more focal labeling where tissue appeared to be spongiotic, although it was difficult to attribute it to a certain cell type due to the mild signal (*1*) and light haematoxylin counterstaining.

No particular deviations from the abovementioned IHC pattern were observed for the VCN. On the other hand, multifocal foci of pericapillary IR (intensity *2–3*; inset in Fig 4b) were apparent in the IC of two old dolphins (ID133, ID95) with evidence of mild microscopic pathology (Table 1). In four animals, a nucleolar IR was particularly evident in the IC external cortex (ID89, ID107, ID145, ID146).

#### Bcl-2

Cytoplasmic IR was scant, and most often detected multifocally in glial cell cytoplasm (*1–3*). In all adults, at least one of the nuclei had negative neuronal cytoplasm (Fig 2d), while all the calves but one (ID 343) displayed mild IR (*1*) in both nuclei.

#### Aβ

In general, Aβ-IR could variably be observed in both the nucleus (*1–3*) and cytoplasm (*1*) of neurons (Fig 2e). A disseminated granular IR (*1–2*) characterized the neuropil and reduced within white matter tracts. Prominent nuclear IR (*3*) appeared multifocally in oligodendrocytes, and a mild astrocytic IR (*1–2*) was evident around the vessels and in spongiotic areas.

With the exceptions of ID142 and ID203, 14 out of the 16 dolphins in which the VCN was available displayed an intense intranuclear IR (*3*) in VCN neurons (inset in Fig 2e). A mildly augmented perineuronal IR was observed in the VCN of putatively older dolphins ID142, ID203 and ID319 (Fig 2e). Additionally, one coalescing plaque (Fig 2f) was detected between the cochlear and superior olivary nuclei of ID319, in whose brain multifocal neuronal lipofuscin deposits were also apparent.

In the IC, nuclear IR was more heterogeneous between the external cortex and core of the structure, with the former displaying stronger intranuclear IR. Neuronal cytoplasmic IR (*1*) was generally weaker compared to that of glial cells.

#### NF 200

A prominent IR (*2–3*) against the cytoskeletal NF200 protein was detected in all visible myelinated axons and perikarya of all tested auditory and non-auditory nuclei (Fig 2g). No qualitative differences were visible between specimens.

**Fig 3:**
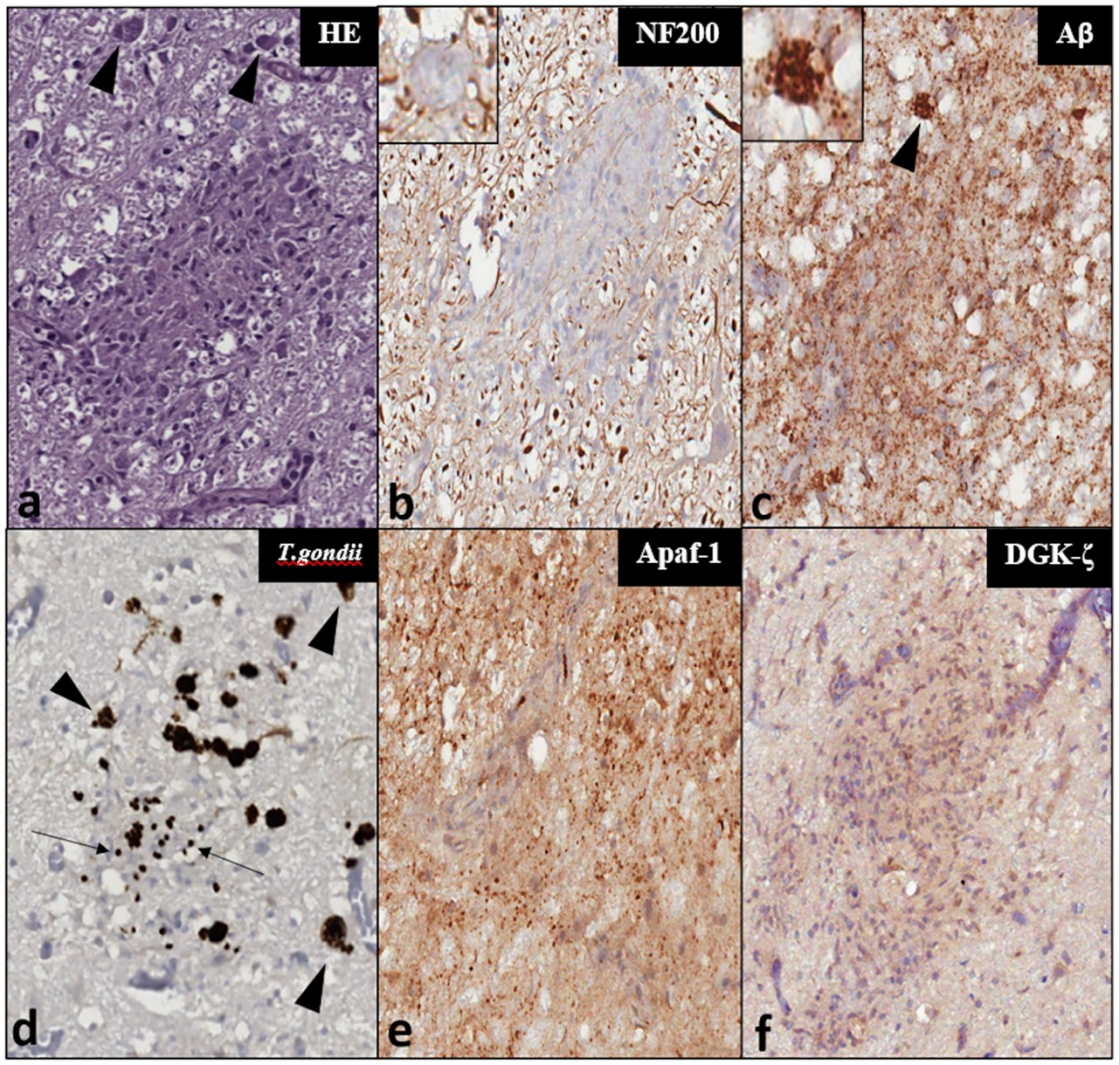
Comparison between histochemical and IHC staining results for a glial node surrounding *T*. *gondii* cysts (arrowheads) in the IC of ID142—Magnification: 200x. Insets display detail of cysts at a magnification of 400x. (a) HE-stain. (b) NF200-IR within the glial node tissue (c) Aβ-IR which also localizes to the *T*. *gondii* cysts (inset); (d) *T*. *gondii* cysts and zoites recognized by a specific anti-*T*. *gondii* antibody; (e) Apaf-1-IR appears attenuated within the cell-rich microglial nodule in comparison to the granular IR and high background of the surrounding neuropil; (f) DGK-ζ-IR is mildly stronger within the glial cells of the parasitic nodule compared to the surrounding neuropil.

**Fig 4:**
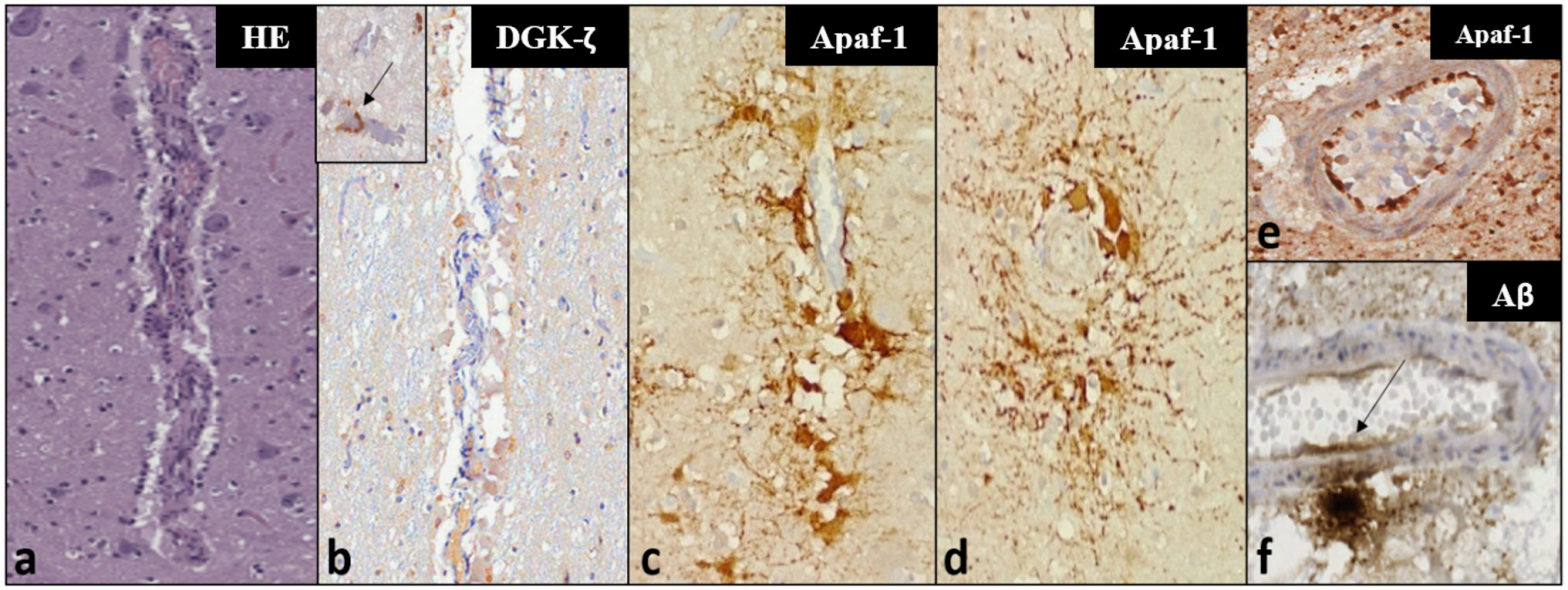
Histochemical (a) and IHC (b-f) findings in vascular and perivascular tissues in the investigated dolphins—Magnification: 100x. Panels a-c represent the same vessel in consecutive sections. (a) Microscopic appearance of a mid-caliber vessel in the IC of ID344 using a haematoxylin-eosin staining, with a mild perivascular infiltrate characterized by mononuclear inflammatory cells. (b) very mild cytoplasmic DGK-ζ-IR in few foci of perivascular glia. Inset: Moderate, multifocal IR in pericapillary neuropil of ID133 (arrow)—magnification: 200x. (c) The same vessel of ID344 displays intense Apaf-1-IR, mostly in the cytoplasm of pericytes, showing perivascular distribution and radiating cellular processes, visible also in transverse sections of the IC vessels of ID344 (d). (e) Clearly demarcated endothelial Apaf-1-IR in a VCN vessel of ID142. (f) Aβ-IR deposit in neuropil adjacent to a mid-caliber VCN vessel—the IR appears to extend into the vessel wall and endothelial lining (arrow).

The most remarkable observed IHC patterns are summarized in Fig 2, while patterns associated with specific inflammatory and vascular phenomena are depicted in Figs 3 and 4, respectively.

**Fig 5:**
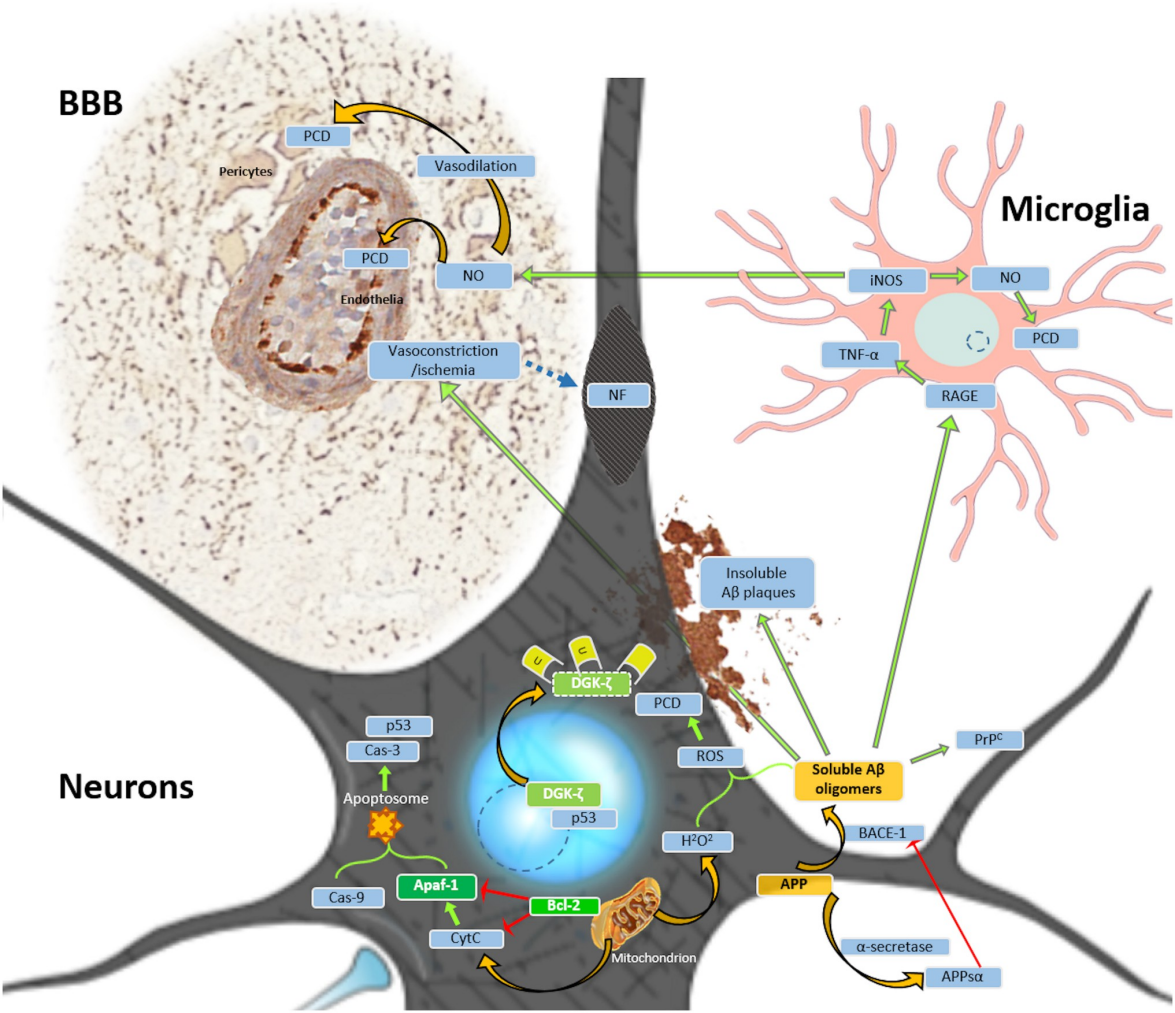
Relevant biological roles of the implemented biomarkers protein in programmed cell death PCD (PCD; green highlight) and Aβ-associated toxicity (orange highlight) as referred to in the main text. Light blue circles—nuclei. Dashed line circles—nucleoli. Orange arrows—translocation and transformation of molecules/processes. Green arrows—activation or induction of molecules. Red arrows—inhibition of molecules. Blue highlight—interacting molecules. Blue dashed arrow: Association between focal ischemia and proteolysis of cytoskeletal neurofilaments (NF), leading to the development of spheroids. Abbreviations: APPsα—soluble APP α; BACE-1—Beta-site APP Cleaving Enzyme 1; BBB—blood brain barrier; Cas—caspase; iNOS—inducible nitric oxide synthase/NOS II; NO—nitric oxide; p53—(tumor) protein 53; Prp^C^—cellular prion protein; RAGE—Receptor for Advanced Glycation End Products; U—ubiquitin (degrades cytoplasmic DGK-ζ). Astrocytes are omitted from this figure for simplification, though they closely interact with both neurons and microglia under both physiological and pathological conditions.

Fig 5 provides a visual overview of the biological roles of the implemented biomarkers.

Even in the heterogeneous sample population available for this study, qualitative combinations of cellular patterns corresponding to certain pathological processes appeared to emerge, although the differences in the patterns of single markers overlapped between these processes.

#### Inflammation

Dolphins with inflammatory lesions (e.g. ID142, ID319, ID165) tended to have stronger cytoplasmic Apaf-1-IR in the astrocytes and surrounding the vasculature, perineuronal Aβ appeared augmented, while intranuclear neuronal Aβ was reduced.

#### Hypoxia

Dolphin ID203, presumed to have asphyxiated in a net, displayed increased cytoplasmic neuronal Apaf-1-IR, a higher ratio of cytoplasmic:nucleolar DGK-ζ-IR, no intranuclear neuronal Aβ and no sign of Bcl-2-IR.

#### Aging

The clearest pattern for old dolphins was the consistent presence of cytoplasmic DGK-ζ-IR in the neurons. In the calves, neuronal Bcl-2 was more frequently observed in the cytoplasm compared to adults.

### Statistical analysis

When the animals were grouped according to microscopic pathological changes in the CNS, significantly higher H-averages (ANOVA; *p = 0*.*0068*) were observed for cytoplasmic Apaf-1 in astrocytes of the IC in adults with evident ongoing CNS pathology (*P*), as opposed to adult animals without visible lesions (*N*). No significant differences emerged between these two groups for the VCN.

Following qualitative IHC assessment of the implemented biomarkers in each dolphin, adults were regrouped according to qualitative deviations from the expected IHC-pattern for a lesion-free brain. In this case, cytoplasmic Apaf-1-IR was significantly higher (ANOVA; *p = 0*.*0067*) in the astrocytes of animals with deviating IHC-patterns (e.g. perivascular IR in Fig 4c–4d).

In the comparison between young (*Y*) and old (*O*) dolphins, a significantly higher nucleolar DGK-ζ-IR was apparent in the IC of young adults (ANOVA; *p = 0*.*0018*), with a similar tendency (*p = 0*.*0786*) apparent for the VCN in the parametric tests, while cytoplasmic DGK-ζ-IR was significantly higher in older adults (ANOVA; *p = 0*.*0330*).

In all three analytical scenarios, statistically significant differences between calves (*C*) and adults resulted from both parametric and non-parametric tests (S1 Table). The most prominent differences were evident for the neuronal cytoplasm in the IC, with the calves yielding relatively higher H-averages for Bcl-2-IR than adults with lesions. A stronger Apaf-1-IR occurred as well in the neuronal cytoplasm compared to both adults with and without CNS pathology in both IC and VCN. Intranuclear neuronal Aβ-IR was significantly more intense and widespread in calves *vs*. both young/old animals, and *vs*. adults with/without evident CNS pathology. Averages of absolute numbers of NF200-IR neurons were higher in calves compared to both adult groups for most analyses.

## Discussion

Understanding the baseline expression of biomarkers within the cetacean CNS is key to future evaluation of animals affected by various insults, including those deriving from suspected overexposure to anthropogenic underwater noise. In the latter case, IHC should be performed on the acoustic pathways, as proposed in the cited literature [7, 26, 43, 44].

Recent protocols attempt to optimize the fixation of cetacean brains for histopathological analyses [23]. Our suggested addition to the CNS sampling protocol (Supplementary Materials) underlines the sampling method for the VCN and IC, which could easily be collected during standard necropsy [5], while also suggesting that one side of the bilateral structures be frozen fresh for future molecular studies going beyond routine pathogen detection (e.g. for Morbillivirus, *Brucella ceti* or *T*. *gondii*). Alternatively, as suggested by Ijsseldijk and colleagues [5], a midline sagittal cut between the two hemispheres and sides of the brainstem could preserve the structural integrity of the hemispheres while allowing fixative to penetrate into the tissue from the ventricles, which would allow potential imaging studies to corroborate microstructural findings. One of the hemispheres could be frozen after taking fresh samples for ancillary diagnostics—serving as a reserve for molecular correlation of histopathological and IHC findings of the fixed half.

The validation process identified the specificity of the IR of five biomarkers of apoptotic (Apaf-1, DGK-ζ, Bcl-2), neurodegenerative (Aβ) and structural (NF200) changes of the neuropil and vasculature, and found them consistent with biomedical literature [7, 26, 33, 43, 44]. These markers were subsequently compared in adult dolphins with and without pathological changes assessed during microscopic examination, and in four calves to account for age-related changes in protein expression.

### Apaf-1

In our study, several markers identifying apoptotic processes have been investigated. Among these, Apaf-1 is the only marker that yielded statistically significant differences for the cytoplasmic astrocyte IR in the IC between dolphins with and without brain lesions in analyses based on pathological and IHC findings. In the literature, Apaf-1 has been shown to activate during early intrinsic apoptosis of various cell types including neuronal [8], glial [45] and endothelial cells [46], inducing apoptosome formation by cleaving initiator caspases [8]. Distinct glial cell types show different sensitivity to Apaf-1-associated PCD [46], and assessment of its expression may enhance our understanding of cetacean CNS pathogenesis.

Apaf-1-IR was more widespread in the cytoplasm of astrocytes of adult dolphins affected by pathologies of the CNS. An increased expression of Apaf-1 in perivascular astrocytes in some dolphins appeared to conflict with the inconspicuous findings of routine histopathology (Fig 4a and 4c). However, the suspected involvement of bacterial toxins in the death of those animals could explain damage spreading from the vasculature [46, 47]. At the same time, Apaf-1-IR appeared to be reduced around glial nodules neighbouring *T*. *gondii* cysts. Therefore, this marker could help evaluate perivascular and glial pathology potentially corresponding to vasoconstriction [48] and toxin exposure [47], without marking the presence or protozoan parasites.

In neurons, a diffuse intranuclear neuronal IR, seen in two older dolphins in this study, could correspond to early apoptotic changes preceding any morphological evidence: it should be noted that intranuclear Apaf-1 concentration has previously been reported to rise significantly in hypoxic laboratory animals [8]. Neuronal cytoplasmic IR was notably less indicative: in fact, the IR in calves was significantly more intense, though how developmental phenomena or hypoxia may influence this remains unclear [49]. It should be noted that all the 9-day-old calves investigated herein died of respiratory distress following meconium aspiration syndrome, likely leading to hypoxic conditions.

Statistically significant differences were absent when comparing neuronal cytoplasmic IR between young (<30 y.o.) and old (≥30 y.o.) adult dolphins. These results imply that Apaf-1 identifies cells affected by noxious stimuli, but does not specifically mark aging cells. Although degenerating neurons are suspected to re-express Apaf-1 in physiological aging, convincing evidence is lacking [50]. It is therefore advisable to consider neonate specimens separately, and to conduct further studies to acquire reliable baseline data for this age group.

### DGK-ζ

DGK-ζ has been reported to undergo irreversible nucleocytoplasmic translocation prior to the cell’s apoptosis in both the cochlear hair cells and various populations of cerebral neurons in mammalian species like guinea pigs and mice [43, 51]. In the present study, statistical analyses for this marker showed significantly higher cytoplasmic- and lower nucleolar IR in older compared to younger adults, implying a higher prevalence of neuronal degeneration in putatively older, stranded animals. Furthermore, It is remarkable that a non-parametric analysis yielded a higher nucleolar DGK-ζ expression bordering on statistical significance in lesion-free dolphins compared to those with microscopically evident brain pathology (S6 Fig).

### Bcl-2

Bcl-2—an established anti-apoptotic marker—is the only one for which neuronal H-averages were unevenly distributed in both IC and VCN. Bcl-2-protein expression has been shown to diminish 15 minutes after acoustic overexposure in mice [7], but there are contradictory reports on its up-/down-regulation [52, 53] under other cellular stress events. Furthermore, quantitative expression was previously reported with molecular techniques like WB, and the present IHC results do not clearly indicate its specificity in the assessment of CNS lesions in bottlenose dolphins.

### Aβ

We next considered a classic indicator of neurodegeneration in brain tissues: Aβ [54–56]. Although it has previously been successfully employed in at least 3 cetacean species [21, 22], to the best of our knowledge, the obtained results were not supported by a validation including WB. Aβ is claimed to target amyloid-β oligomer 1–42, which is found in cytotoxic extracellular plaques in human Alzheimer’s disease (AD) [56]. In our study, WB analyses yielded clear IR bands for all examined cetacean species at 87 kDa—a molecular weight corresponding to one of the APP isoforms [55].

In our study, the diffuse granular IR in the neuropil of the majority of the investigated dolphins could be justified, considering that APP yields a variety of cleavage products. Examples range from cytoprotective APPsα to Aβ1–42. The latter, generated via the amyloidogenic pathway, induces neurotoxicity through metabolic disruption and induction of apoptosis [56]. Intranuclear neuronal localization of this Aβ isoform has previously been reported in the brains of 2 out of 9 animals belonging to two cetacean species (pantropical spotted dolphin, *Stenella frontalis*, and BW) [22]. Despite using a higher antibody dilution in our study (1:1000 *vs*. 1:200), intranuclear neuronal IR was evident in at least one of the two auditory nuclei in all investigated specimens. The reasons could include species-specific functional specializations [57], and the higher intensity of the heat-induced antigen retrieval method used by the semi-automatic immunostainer used in our study. A higher initial demasking may have exposed more of the antigen, making it more available for the antibody, even at a higher dilution of the latter. The intranuclear location-associated role of this protein is under debate, with several neurodegenerative diseases associated with intranuclear Aβ aggregates [58]. Other studies suggest a neuroprotective role, such as against hypoxia [22, 56]. Aβ 1–42 is known to inhibit Apaf-1-induced intrinsic apoptosis, albeit to a limited degree [59]. The light IR observed in the cytoplasm of both glial and neuronal cells could therefore also represent an adaptation of an organism evolved to buffer the consequences of hypoxia in all its tissues including the CNS, as documented by comparatively higher anti-oxidant protein levels in cetacean brains [60], and a higher degree of myelination in deeper diving species [57].

As with DGK-ζ, if one does not assume normal distribution, young adults and calves display more intranuclear Aβ than older adults. Investigating a larger sample population and employing antibodies against Aβ-interactors, such as PrP^**c**^ [61], would be recommended to understand the meaning of this diminution in aged individuals.

The presence of a coalescing extracellular amyloid plaque in the white matter between the VCN and the superior olivary complex of an older bottlenose dolphin is arguably the most interesting finding of the herein reported microscopic investigations. Amyloid plaques have previously been reported in the human central auditory system [62]. In this study, the authors reasoned that impaired connectivity in major integration centers, such as the IC, could well contribute to the auditory impairment seen in many AD-affected patients. Our evidence does not support or suggest that amyloid plaques may impact the sensory abilities of the dolphin. However, connections between AD-associated neurovascular pathology and age-related hearing loss are consistently reported in the human literature [63]. Neurodegenerative diseases may also impact cetaceans because of the extended life span of these aquatic mammals. In this sense, dolphins and whales may hold potential as comparative translational models for the onset, development and evolution of human AD [20]. Perivascular Aβ-IR enhancement is also important to consider for the regulation of blood vessel caliber. The downstream interactions with TNFα [16], released by microglial cells interacting with Aβ through their RAGE (*Receptor for Advanced Glycation End Products*) receptors [64], may further promote iNOS biosynthesis, with subsequent production of nitric oxide (NO), a powerful vasodilator [31]. Conversely, Aβ has also been reported to cause a marked vasoconstriction [30]. In large quantities, NO may induce apoptosis [31]. Thus, comparing perivascular Aβ- and Apaf-1-IR may facilitate assessment of vascular pathology.

### NF 200

Concerning structural changes, NF200 is considered to be a reliable cytoskeletal marker to visualize neuronal structure. Our preliminary density estimates did not yield significant differences between dolphins with/without evident CNS pathology, although NF200 has been reported to be downregulated in studies on hypoxic-ischemic and traumatic brain injury, with the presence of spheroids marking the loss of structural integrity [32, 33]. Nevertheless, further studies (e.g. using co-labeling) are needed before making inferences on the pathological effects on NF200 expression. Finally, the lack of significant differences among adult animals, coupled with the simultaneous presence of statistically significant differences in the expression of pro-apoptotic/apoptotic biomarkers, underline the importance of IHC-based investigations on these biomarkers to assess tissue damage before microscopic lesions become apparent.

Despite these encouraging results, several limitations were identified and considered. Some of the investigated biomarkers were negative according to one of the orthogonal methods of validation, such as Apaf-1 in the WB analysis. While its specificity in the presently reported IHC staining was convincing, the percentage of amino acid sequence homology for the protein targeted by the tested antibody was moderate (85.83%). Moreover, the protein sequence was predicted from the genomic level via automated computational analysis, which may harbor errors by not considering species-specific post-transcriptional modifications. Further studies including independent epitope validation [64] should be performed to corroborate these results. NF200-IR in the WB was very weak for bottlenose and striped dolphins, but very pronounced for fin, BW and sperm whales. However, clear-cut IHC specificity of this biomarker for the cytoskeleton of perikarya and axons was found in this and in previous studies [65].

Due to the extremely delicate nature of the CNS, differentiating *post mortem* changes from *ante mortem* lesions is crucial. The examined brains were sampled from DCC 1–2 animals and the fixation procedure was aimed at preserving the tissue for cytoarchitectural studies [36, 37], being consistent through the entire study. Furthermore, where clear evidence of *post mortem* changes (gas bubbles, enlargement of Virchow-Robin spaces, bacterial presence etc.) was apparent, no IR enhancement for any of the five studied biomarkers could be detected. In a previous study, we established that using the same protocols in canine brains with *post mortem* intervals ranging up to 72 hours, these biomarkers did not show significant IR pattern/intensity changes [66]. However, the blubber layer of cetaceans tends to induce a stronger increase in *post mortem* temperature, accelerating autolysis. It remains vital to examine only very well preserved carcasses.

Calves were also included in our study to account for the likelihood of protein expression differences secondary to developmental processes occurring in neonatal brains. Although the stronger Bcl-2-IR in the neuronal cytoplasm and the more consistent DGK-ζ-IR in neuronal nucleoli could be indicative of tissues more resistant to apoptosis [67], this does not imply that a significant neuroprotective mechanism in animals at this developmental stage represents the only explanation.

## Conclusion

The biomarkers validated in this study promise additional diagnostic information for microscopic analysis of the CNS, particularly for pre- to early-apoptotic (Apaf-1, DGK-ζ), but also neurodegenerative, inflammatory, and even reversible vascular changes (Aβ) [30]. Apaf-1-IR yielded additional qualitative evidence of neurochemical dysregulation, a feature elusive in routine microscopic analysis, differing significantly between adult dolphins with and without CNS-lesions. DGK-ζ-IR appeared more shifted to neuronal cytoplasm than to the nucleolus in older adults. Aβ, due to its variable IR, requires further investigation regarding its function and reflection of evolutionary adaptations in cetaceans. Structural changes (NF200) were not observed, but further studies implementing 3-dimensional advanced microscopy may provide different insights. The fact that these antibodies helped visualize cellular processes evasive in a purely morphological analysis implies their particular usefulness in cases of suspected noise overexposure in marine mammals, especially for subtle cases that might exhibit cumulative damage rather than extreme cases where decompression sickness-like symptoms prevail and inner ear lesions are evident.

It is noteworthy that only 5 out of the 12 tested markers actually passed the hurdles of systematic validation, which underlines that we cannot rely only on IHC results of antibodies that were not custom-made for dolphins. Future analyses must justify their use of antibodies with rigorous scrutiny of the markers they use.

Laboratory animal studies imply temporally differentiated expression profiles of the investigated biomarkers in auditory structures after acoustic overexposure. Systematically validating and applying IHC biomarkers in the VCN and IC of cetacean brains could therefore help answer the question of how much time passed between a potentially anthropogenic acoustic trauma and a mass stranding of cetaceans.

Exposure to environmental pollutants may result in the occurrence of central neuropathies in other marine mammals, as in domoic acid-intoxicated California sea lions (*Zalophus californianus*) [2], or methylmercury and cyanotoxin-exposed short-beaked common dolphins (*Delphinus delphis*) [4] thus other forms of pollution can also be characterized. Additionally, long-lived marine mammals could serve as comparative pathology models for human neurological diseases [20].

Finally, understanding *in situ* protein expression is a vital step in bridging the gap between currently available research techniques and future establishment of organoid models for various types of pathology [68]. Therefore, expanding the preliminary baseline reference data reported herein and understanding interactions between the markers, but also applying quantifiable biomolecular techniques will be crucial to understand pathophysiological processes in cetaceans.

## Supporting information

S1 Fig Age-length estimation curve for the tested bottlenose dolphins. (TIF)

S2 Fig Shapiro-Wilks tests for normality. (TIF)

S3 Fig Raw images of the Western blot membranes for DGK-ζ, Bcl-2, Aβ and NF200 antibodies, captured with the iBright machine (ThermoFisherScientific) and used in Fig 1. The blue “X” marks the lanes and rows that are not included in the final image. Nota bene: The 9 left lanes of these blots correspond to species not included in this study.
(ZIP)

S4 Fig Apaf-1-IR localizes to Nissl-substance in large pyramidal neurons of ID343. Other neurons display an agranularcytoplasmic IR (intensity score 2). Magnification 400x.
(TIF)

S5 Fig Acellular perivascular Apaf-1 positivity in IC of ID201. Magnification 400x.
(TIF)

S6 Fig Box-plot demonstrating the difference in the histo score average between dolphins brains classified according to their IHC patterns. (TIF)

S7 Fig Overview of histopathological CNS-lesions visible in hematoxylin-eosin slides of the dolphins in this study. (ZIP)

S1 Table Results of the statistical comparisons of groups made according to A) morphopathological findings in haematoxylin-eosin stained sections, B) immunohistochemical findings C) age. In gray—results that do not fit into the parametric/non-parametric category according to results of the Shapiro-Wilks and/or Levene’s tests. If the group differences persist in post-hoc analyses, level of adjustment for α-error are documented. Bf—Bonferroni adjustment for α-error.
(PDF)

S2 Table Summary of the ID, sex, body length, full histopathological findings of the CNS, and the most probable cause of death of the dolphins included in this study. (PDF)

## References

[1] Sierra E, Fernández A, Felipe-Jiménez I, Zucca D, Di Francesco G, Díaz-Delgado J, et al. Neurobrucellosis in a common bottlenose dolphin (Tursiops truncatus) stranded in the Canary Islands. BMC Vet Res. 2019;15: 1–8. 31638986 10.1186/s12917-019-2089-0 PMC6805616

[2] Silvagni PA, Lowenstine LJ, Spraker T, Lipscomb TP, Gulland FMD. Pathology of domoic acid toxicity in California sea lions (Zalophus californianus). Vet Pathol. 2005;42: 184–191. doi: 10.1354/vp.42-2-184 15753472

[3] Morell M, Brownlow A, McGovern B, Raverty SA, Shadwick RE, André M. Implementation of a method to visualize noise-induced hearing loss in mass stranded cetaceans. Sci Rep. 2017;7: 1–8. 28165504 10.1038/srep41848 PMC5292969

[4] Davis DA, Garamszegi SP, Banack SA, Dooley PD, Coyne TM, McLean DW, et al. Bmaa, methylmercury, and mechanisms of neurodegeneration in dolphins: A natural model of toxin exposure. Toxins. 2021. doi: 10.3390/toxins13100697 34678990 PMC8540894

[5] Ijsseldijk LL, Brownlow AC, Mazzariol S. Best practice on cetacean post mortem investigation and tissue sampling. 2019.

[6] Summers B. Veterinary neuropathology. St. Louis Mo.: Mosby; 1995.

[7] Gröschel M, Basta D, Ernst A, Mazurek B, Szczepek AJ. Acute noise exposure is associated with intrinsic apoptosis in murine central auditory pathway. Front Neurosci. 2018;12: 1–14. 29867323 10.3389/fnins.2018.00312 PMC5954103

[8] Ashraf QM, Mishra OP, Delivoria-Papadopoulos M. Mechanisms of expression of apoptotic protease activating factor-1 (Apaf-1) in nuclear, mitochondrial and cytosolic fractions of the cerebral cortex of newborn piglets. Neurosci Lett. 2007;415: 253–258. doi: 10.1016/j.neulet.2007.01.023 17275190 PMC1892182

[9] Goto K, Tanaka T, Nakano T, Okada M, Hozumi Y, Topham MK, et al. DGKζ under stress conditions: “To be nuclear or cytoplasmic, that is the question”. Adv Biol Regul. 2014;54: 242–253. doi: 10.1016/j.jbior.2013.08.007 24119575

[10] Di Guardo G, Di Francesco CE, Eleni C, Cocumelli C, Scholl F, Casalone C, et al. Morbillivirus infection in cetaceans stranded along the Italian coastline: Pathological, immunohistochemical and biomolecular findings. Res Vet Sci. 2013;94: 132–137. doi: 10.1016/j.rvsc.2012.07.030 22921372

[11] Díaz-Delgado J, Fernández A, Sierra E, Sacchini S, Andrada M, Vela AI, et al. Pathologic findings and causes of death of stranded cetaceans in the Canary Islands (2006–2012). PLoS ONE. 2018. doi: 10.1371/journal.pone.0204444 30289951 PMC6173391

[12] Henderson D, Bielefeld EC, Harris KC, Hu BH. The role of oxidative stress in noise-induced hearing loss. Ear and Hearing. 2006. pp. 1–19. doi: 10.1097/01.aud.0000191942.36672.f3 16446561

[13] Di Filippo M, Sarchielli P, Picconi B, Calabresi P. Neuroinflammation and synaptic plasticity: theoretical basis for a novel, immune-centred, therapeutic approach to neurological disorders. Trends in Pharmacological Sciences. Elsevier Current Trends; 2008. pp. 402–412. doi: 10.1016/j.tips.2008.06.005 18617277

[14] Panganiban CH, Barth JL, Darbelli L, Xing Y, Zhang J, Li H, et al. Noise-induced dysregulation of Quaking RNA binding proteins contributes to auditory nerve demyelination and hearing loss. J Neurosci. 2018;38: 2551–2568. doi: 10.1523/JNEUROSCI.2487-17.2018 29437856 PMC5858596

[15] Gama Sosa MA, De Gasperi R, Pryor D, Perez Garcia GS, Perez GM, Abutarboush R, et al. Low-level blast exposure induces chronic vascular remodeling, perivascular astrocytic degeneration and vascular-associated neuroinflammation. Acta Neuropathol Commun. 2021;9: 1–27. 34654480 10.1186/s40478-021-01269-5 PMC8518227

[16] Wang W, Zhang LS, Zinsmaier AK, Patterson G, Leptich EJ, Shoemaker SL, et al. Neuroinflammation mediates noise-induced synaptic imbalance and tinnitus in rodent models. PLoS Biol. 2019;17: e3000307. doi: 10.1371/journal.pbio.3000307 31211773 PMC6581239

[17] Glezer II, Hof PR, Morgane PJ. Comparative analysis of calcium-binding protein-immunoreactive neuronal populations in the auditory and visual systems of the bottlenose dolphin (Tursiops truncatus) and the macaque monkey (Macaca fascicularis). 1998;15: 203–237. 10.1016/s0891-0618(98)00022-2 9860088

[18] Hof PR, Glezer II, Condé F, Flagg RA, Rubin MB, Nimchinsky EA, et al. Cellular distribution of the calcium-binding proteins parvalbumin, calbindin, and calretinin in the neocortex of mammals: Phylogenetic and developmental patterns. J Chem Neuroanat. 1999;16: 77–116. doi: 10.1016/s0891-0618(98)00065-9 10223310

[19] Cozzi B, Roncon G, Granato A, Giurisato M, Castagna M, Peruffo A, et al. The claustrum of the bottlenose dolphin Tursiops truncatus (Montagu 1821). Front Syst Neurosci. 2014;8: 1–8. 24734007 10.3389/fnsys.2014.00042 PMC3975097

[20] Gunn-Moore D, Kaidanovich-Beilin O, Gallego Iradi MC, Gunn-Moore F, Lovestone S. Alzheimer’s disease in humans and other animals: A consequence of postreproductive life span and longevity rather than aging. Alzheimer’s Dement. 2018;14: 195–204. 28972881 10.1016/j.jalz.2017.08.014

[21] Stylianaki I, Komnenou AT, Posantzis D, Nikolaou K, Papaioannou N. Alzheimer’s disease-like pathological lesions in an aged bottlenose dolphin (Tursiops truncatus). Vet Rec Case Reports. 2019;7: 1–5. doi: 10.1136/vetreccr-2018-000700

[22] Simona S, Antonio EDLM, Yania P. Amyloid-beta peptide and phosphorylated tau in the frontopolar cerebral cortex and in the cerebellum of toothed whales: aging vs hypoxia Summary Statement: Biology Open • Accepted manuscript. 10.1242/bio.054734 PMC7657478 33037014

[23] Sacchini S, Herráez P, Arbelo M, Espinosa de los Monteros A, Sierra E, Rivero M, et al. Methodology and Neuromarkers for Cetaceans’ Brains. Vet Sci. 2022;9: 1–19. doi: 10.3390/vetsci9020038 35202291 PMC8879147

[24] Mazzariol S, Centelleghe C, Cozzi B, Povinelli M, Marcer F, Ferri N, et al. Multidisciplinary studies on a sick-leader syndrome-associated mass stranding of sperm whales (Physeter macrocephalus) along the Adriatic coast of Italy. Sci Rep. 2018;8: 1–18. 30068967 10.1038/s41598-018-29966-7 PMC6070578

[25] Ridgway SH. The Auditory Central Nervous System of Dolphins. Springer, New York, NY; 2000. pp. 273–293.

[26] Morell M, IJsseldijk LL, Piscitelli-Doshkov M, Ostertag S, Estrade V, Haulena M, et al. Cochlear apical morphology in toothed whales: Using the pairing hair cell—Deiters’ cell as a marker to detect lesions. Anat Rec. 2021 [cited 1 Jul 2021]. doi: 10.1002/ar.24680 34096183

[27] Choi SH, Choi CH. Noise-induced neural degeneration and therapeutic effect of antioxidant drugs. Korean J Audiol. 2015;19: 111–119. doi: 10.7874/jao.2015.19.3.111 26771008 PMC4704551

[28] Abbott SD, Hughes LF, Bauer CA, Salvi R, Caspary DM. Detection of glutamate decarboxylase isoforms in rat inferior colliculus following acoustic exposure. Neuroscience. 1999;93: 1375–1381. doi: 10.1016/s0306-4522(99)00300-0 10501462

[29] Tamagno E, Parola M, Guglielmotto M, Santoro G, Bardini P, Marra L, et al. Multiple signaling events in amyloid β-induced, oxidative stress-dependent neuronal apoptosis. Free Radic Biol Med. 2003;35: 45–58. doi: 10.1016/s0891-5849(03)00244-2 12826255

[30] Nortley R, Korte N, Izquierdo P, Hirunpattarasilp C, Mishra A, Jaunmuktane Z, et al. Amyloid b oligomers constrict human capillaries in Alzheimer’s disease via signaling to pericytes. Science (80-). 2019;365. doi: 10.1126/science.aav9518 31221773 PMC6658218

[31] Fabrizi C, Silei V, Menegazzi M, Salmona M, Bugiani O, Tagliavini F, et al. The Stimulation of Inducible Nitric-oxide Synthase by the Prion Protein Fragment 106–126 in Human Microglia is Tumor Necrosis Factor-α-dependent and Involves p38 Mitogen-activated Protein Kinase. J Biol Chem. 2001;276: 25692–25696. doi: 10.1074/jbc.M100133200 11316802

[32] Posmantur R, Hayes RL, Dixon CE, Taft WC. Neurofilament 68 and Neurofilament 200 Protein Levels Decrease After Traumatic Brain Injury. J Neurotrauma. 1994;11: 533–545. doi: 10.1089/neu.1994.11.533 7861446

[33] Mages B, Aleithe S, Altmann S, Blietz A, Nitzsche B, Barthel H, et al. Impaired neurofilament integrity and neuronal morphology in different models of focal cerebral ischemia and human stroke tissue. Front Cell Neurosci. 2018;12: 1–15. 29967576 10.3389/fncel.2018.00161 PMC6015914

[34] Kraus KS, Ding D, Jiang H, Lobarinas E, Sun W, Salvi RJ. Relationship between noise-induced hearing-loss, persistent tinnitus and growth-associated protein-43 expression in the rat cochlear nucleus: Does synaptic plasticity in ventral cochlear nucleus suppress tinnitus? Neuroscience. 2011;194: 309–325. doi: 10.1016/j.neuroscience.2011.07.056 21821100 PMC3390756

[35] K SG, L MC. Acceleration of age-related hearing loss by early noise exposure: evidence of a misspent youth. J Neurosci. 2006;26: 2115–2123. doi: 10.1523/JNEUROSCI.4985-05.2006 16481444 PMC1855187

[36] Ballarin C, Papini L, Bortolotto A, Butti C, Peruffo A, Mazzariol S. An on-line tissue bank for marine mammals of the Mediterranean sea and adjacent waters. Hystrix—Ital J Mammal. 2006;16. doi: 10.4404/hystrix-16.2-4350

[37] Graïc JM, Peruffo A, Grandis A, Cozzi B. Topographical and structural characterization of the V1–V2 transition zone in the visual cortex of the long-finned pilot whale Globicephala melas (Traill, 1809). Anat Rec. 2021;304: 1105–1118. doi: 10.1002/AR.24558 33119932

[38] De Vreese S, André M, Cozzi B, Centelleghe C, Van Der Schaar M, Mazzariol S. Morphological Evidence for the Sensitivity of the Ear Canal of Odontocetes as shown by Immunohistochemistry and Transmission Electron Microscopy. 2020; 1–17. doi: 10.1038/s41598-020-61170-4 32144309 PMC7060263

[39] Ramos-Vara J, Kiupel M, Baszler T, Bliven L, Brodersen B, Chelack B, et al. Suggested guidelines for immunohistochemical techniques in veterinary diagnostic laboratories. 2008;413: 393–413. 10.1177/104063870802000401 18599844

[40] MacNeil T, Vathiotis IA, Martinez-Morilla S, Yaghoobi V, Zugazagoitia J, Liu Y, et al. Antibody validation for protein expression on tissue slides: A protocol for immunohistochemistry. BioTechniques. Future Science Ltd; 2020. pp. 461–468. doi: 10.2144/btn-2020-0095 32852223 PMC7807291

[41] Mazzariol S, Centelleghe C, Petrella A, Marcer F, Beverelli M, Di Francesco CE, et al. Atypical Toxoplasmosis in a Mediterranean Monk Seal (Monachus monachus) Pup. J Comp Pathol. 2021;184: 65–71. doi: 10.1016/j.jcpa.2021.02.005 33894880

[42] Mazzariol S, Cozzi B, Centelleghe C. Handbook for Cetaceans’ Strandings. 2015; 240.

[43] Shinkawa C, Ito T, Hozumi Y, Chiba M, Matsui H, Goto K, et al. Expression and localization of diacylglycerol kinase ζ in guinea pig cochlea and its functional implication under noise-exposure stress conditions. Histochem Cell Biol. 2019;151: 461–474. doi: 10.1007/s00418-019-01781-9 30963236

[44] Pun PBL, Kan EM, Salim A, Li Z, Ng KC, Moochhala SM, et al. Low level primary blast injury in rodent brain. Front Neurol. 2011;APR: 1–15. 21541261 10.3389/fneur.2011.00019 PMC3083909

[45] Rose J, Brian C, Woods J, Pappa A, Panayiotidis MI, Powers R, et al. Mitochondrial dysfunction in glial cells: Implications for neuronal homeostasis and survival. Toxicology. 2017;391: 109–115. doi: 10.1016/j.tox.2017.06.011 28655545 PMC5681369

[46] Chong ZZ, Kang JQ, Maiese K. Apaf-1, Bcl-xL, cytochrome c, and caspase-9 form the critical elements for cerebral vascular protection by erythropoietin. J Cereb Blood Flow Metab. 2003;23: 320–330. doi: 10.1097/01.WCB.0000050061.57184.AE 12621307

[47] Berrich M, Boulouis H-J, Monteil M, Kieda C, Haddad N. Vascular Endothelium and Vector Borne Pathogen Interactions. Curr Immunol Rev. 2012;8: 227–247. doi: 10.2174/157339512800672010

[48] Intengan HD, Schiffrin EL. Vascular remodeling in hypertension. Hypertens Princ Pract. 2001;Hypertensi: 581–587. doi: 10.1161/hy09t1.096249 11566935

[49] Yoshida H, Kong Y, Yoshida R, Elia AJ, Hakem A, Hakem R, et al. Apaf1 Is Required for Mitochondrial Pathways of Apoptosis and Brain Development. 1998;94: 739–750. 10.1016/s0092-8674(00)81733-x 9753321

[50] Kole AJ, Annis RP, Deshmukh M. Mature neurons: equipped for survival. Cell Death Dis. 2013;4: 1–8. doi: 10.1038/cddis.2013.220 23807218 PMC3702294

[51] Okada M, Hozumi Y, Tanaka T, Suzuki Y, Yanagida M, Araki Y, et al. DGK ζ is degraded through the cytoplasmic ubiquitin—proteasome system under excitotoxic conditions, which causes neuronal apoptosis because of aberrant cell cycle reentry. Cell Signal. 2012;24: 1573–1582. doi: 10.1016/j.cellsig.2012.03.021 22516102

[52] Siddiqui WA, Ahad A, Ahsan H. The mystery of BCL2 family: Bcl-2 proteins and apoptosis: an update. Arch Toxicol. 2015;89: 289–317. doi: 10.1007/s00204-014-1448-7 25618543

[53] Shinoura N, Yoshida Y, Nishimura M, Muramatsu Y, Asai A, Kirino T, et al. Expression level of Bcl-2 determines anti- or proapoptotic function. Cancer Res. 1999;59: 4119–4128. 10463617

[54] Querfurth HW, Laferla FM. Alzheimer’s Disease. 2010; 329–344. 10.1056/NEJMra0909142 20107219

[55] Bros P, Delatour V, Vialaret J, Lalere B, Barthelemy N, Gabelle A, et al. Quantitative detection of amyloid-β peptides by mass spectrometry: State of the art and clinical applications. Clinical Chemistry and Laboratory Medicine. Walter de Gruyter GmbH; 2015. pp. 1483–1493. doi: 10.1515/cclm-2014-1048 25719328

[56] Hefter D, Draguhn A. APP as a protective factor in acute neuronal insults. Frontiers in Molecular Neuroscience. Frontiers Media S.A.; 2017. p. 22. doi: 10.3389/fnmol.2017.00022 28210211 PMC5288400

[57] Ridgway SH, Brownson RH, van Alstyne KR, Hauser RA. Higher neuron densities in the cerebral cortex and larger cerebellums may limit dive times of delphinids compared to deep-diving toothed whales. PLoS One. 2019;14: e0226206. doi: 10.1371/journal.pone.0226206 31841529 PMC6914331

[58] von Mikecz A. Pathology and function of nuclear amyloid: Protein homeostasis matters. Nucleus (United States). Landes Bioscience; 2014. doi: 10.4161/nucl.29404 25482120 PMC4152345

[59] Sharoar MG, Islam MI, Shahnawaz M, Shin SY, Park IS. Amyloid β binds procaspase-9 to inhibit assembly of Apaf-1 apoptosome and intrinsic apoptosis pathway. Biochim Biophys Acta—Mol Cell Res. 2014;1843: 685–693. doi: 10.1016/j.bbamcr.2014.01.008 24424093

[60] Cantú-Medellín N, Byrd B, Hohn A, Vázquez-Medina JP, Zenteno-Savín T. Differential antioxidant protection in tissues from marine mammals with distinct diving capacities. Shallow/short vs. deep/long divers. Comp Biochem Physiol—A Mol Integr Physiol. 2011;158: 438–443. doi: 10.1016/j.cbpa.2010.11.029 21147244

[61] Di Guardo G. Cetaceans, models for human disease? Research in Veterinary Science. Elsevier B.V.; 2018. pp. 43–44. doi: 10.1016/j.rvsc.2018.05.012 29804053

[62] Ohm TG, Braak H. Auditory brainstem nuclei in Alzheimer’s disease. Neurosci Lett. 1989;96: 60–63. doi: 10.1016/0304-3940(89)90243-7 2648201

[63] Shityakov S, Hayashi K, Störk S, Scheper V, Lenarz T, Förster CY. The conspicuous link between ear, brain and heart–could neurotrophin-treatment of age-related hearing loss help prevent alzheimer’s disease and associated amyloid cardiomyopathy? Biomolecules. 2021;11. doi: 10.3390/biom11060900 34204299 PMC8235707

[64] Laurén J, Gimbel DA, Nygaard HB, Gilbert JW, Strittmatter SM. Cellular prion protein mediates impairment of synaptic plasticity by amyloid-Β oligomers. Nature. 2009;457: 1128–1132. doi: 10.1038/nature07761 19242475 PMC2748841

[65] Morell M, Raverty SA, Mulsow J, Haulena M, Barrett-Lennard L, Nordstrom CA, et al. Combining Cochlear Analysis and Auditory Evoked Potentials in a Beluga Whale With High-Frequency Hearing Loss. Front Vet Sci. 2020;7: 534917. doi: 10.3389/fvets.2020.534917 33330679 PMC7672125

[66] Orekhova K, Mazzariol S, Sussan B, Bucci M, Bonsembiante F, Verin R, et al. Immunohistochemical Markers of Apoptotic and Hypoxic Damage Facilitate Evidence-Based Assessment in Pups with Neurological Disorders. Vet Sci 2021, Vol 8, Page 203. 2021;8: 203. doi: 10.3390/vetsci8100203 34679033 PMC8537515

[67] Bernier PJ, Parent A. Bcl-2 protein as a marker of neuronal immaturity in postnatal primate brain. J Neurosci. 1998;18: 2486–2497. doi: 10.1523/JNEUROSCI.18-07-02486.1998 9502809 PMC6793102

[68] Sun AX, Ng HH, Tan EK. Translational potential of human brain organoids. Ann Clin Transl Neurol. 2018;5: 226–235. doi: 10.1002/acn3.505 29468184 PMC5817829

