# Supplementary figures and images for: Systematic validation and assessment of immunohistochemical markers for central nervous system pathology in cetaceans, with emphasis on auditory pathways
Source: PLoS One. 2022 Jun 1;17(6):e0269090. doi: 10.1371/journal.pone.0269090 (PMC9159615; doi:10.1371/journal.pone.0269090)

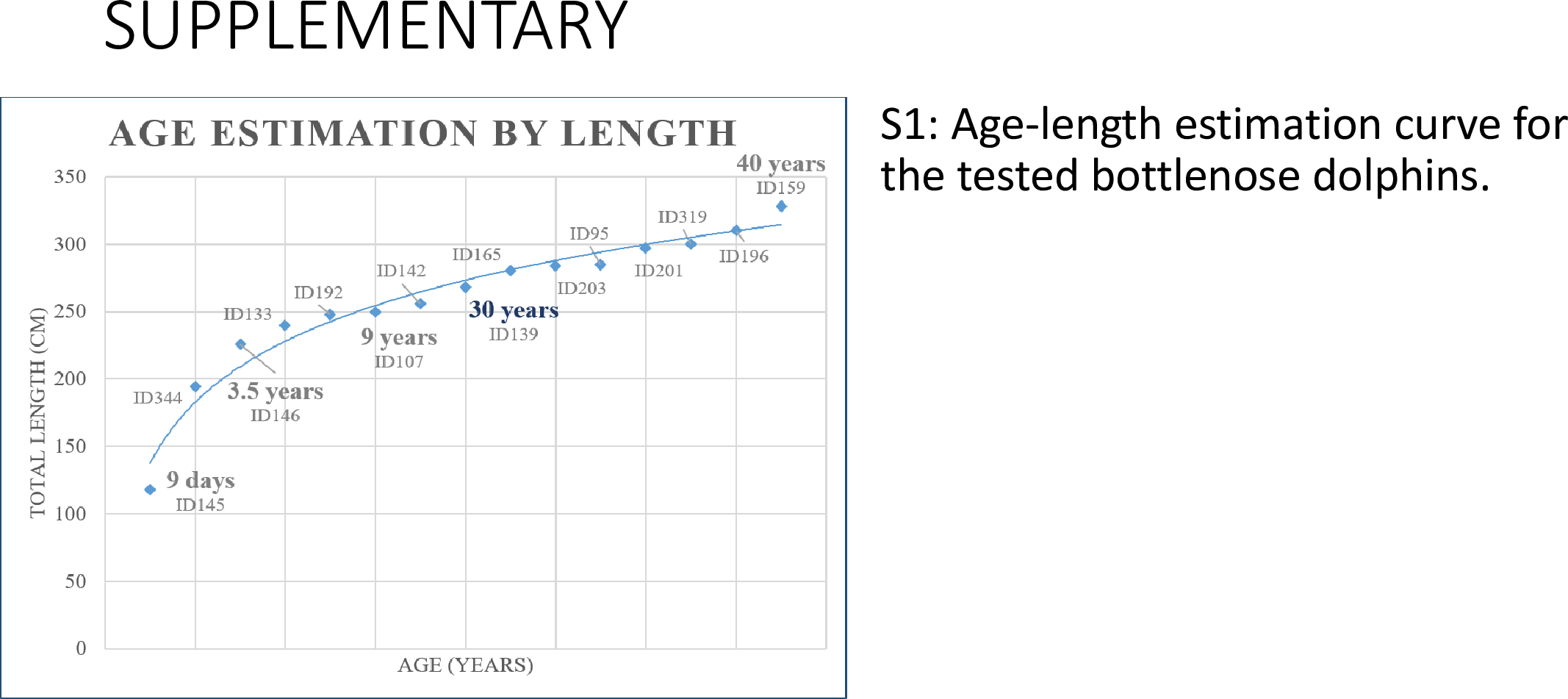

Supplement: S1 Fig — (TIF) [file pone.0269090.s001.tif]

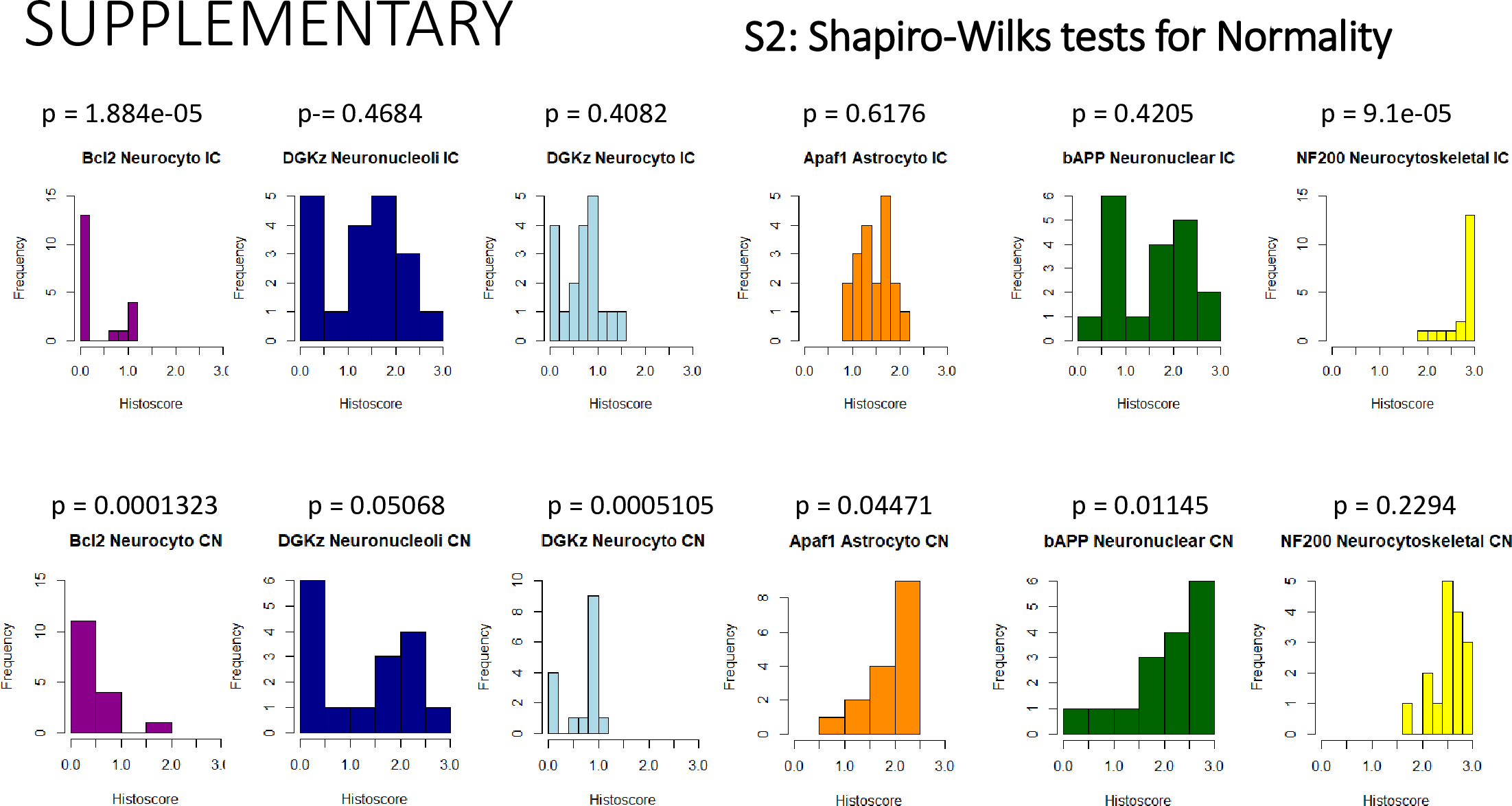

Supplement: S2 Fig — (TIF) [file pone.0269090.s002.tif]

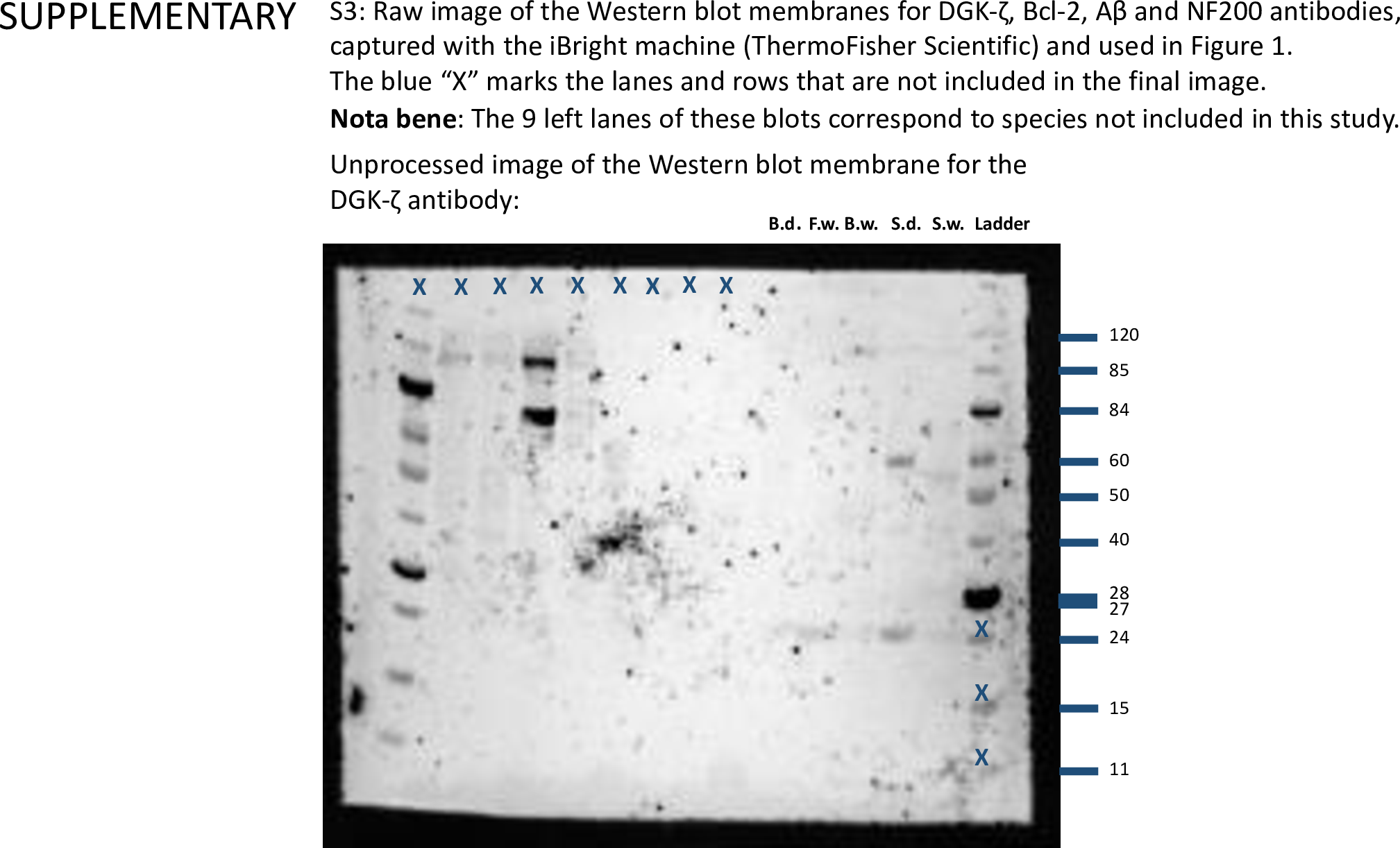

Supplement: S3 Fig — The blue “X” marks the lanes and rows that are not included in the final image. Nota bene: The 9 left lanes of these blots correspond to species not included in this study. (ZIP) [file pone.0269090.s003.zip › Supplementary_FigS3_1.tif]

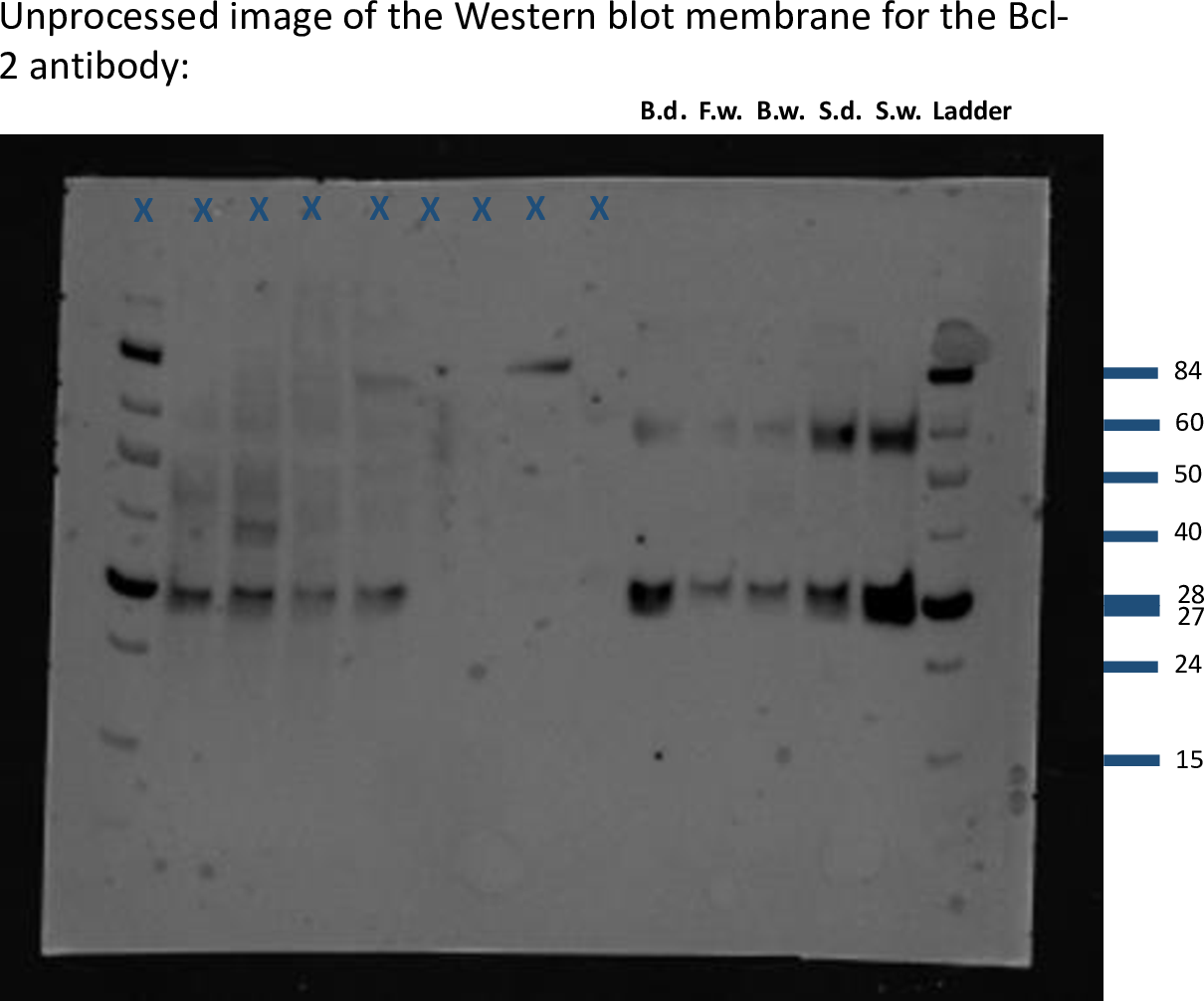

Supplement: S3 Fig — The blue “X” marks the lanes and rows that are not included in the final image. Nota bene: The 9 left lanes of these blots correspond to species not included in this study. (ZIP) [file pone.0269090.s003.zip › Supplementary_FigS3_2.tif]

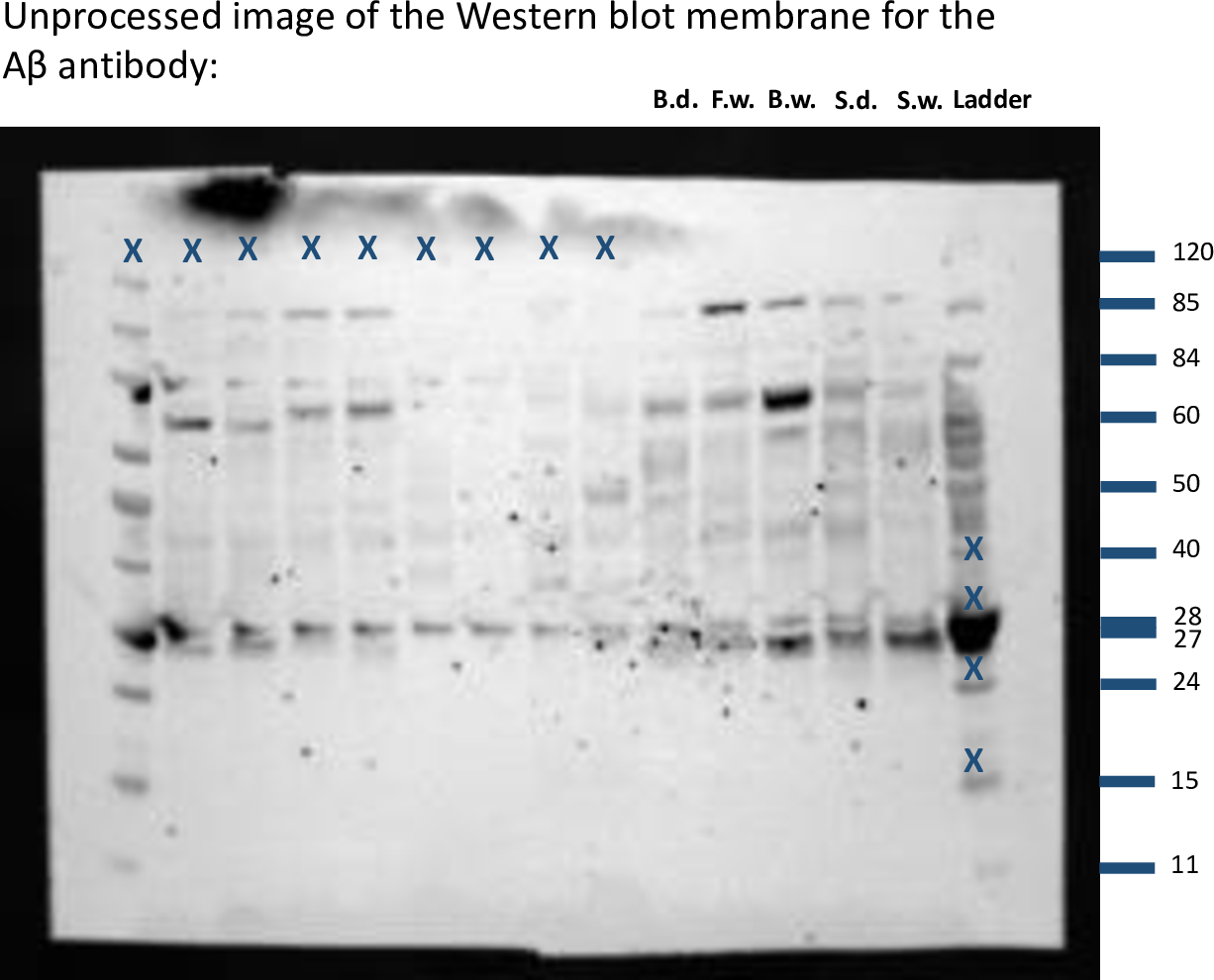

Supplement: S3 Fig — The blue “X” marks the lanes and rows that are not included in the final image. Nota bene: The 9 left lanes of these blots correspond to species not included in this study. (ZIP) [file pone.0269090.s003.zip › Supplementary_FigS3_3.tif]

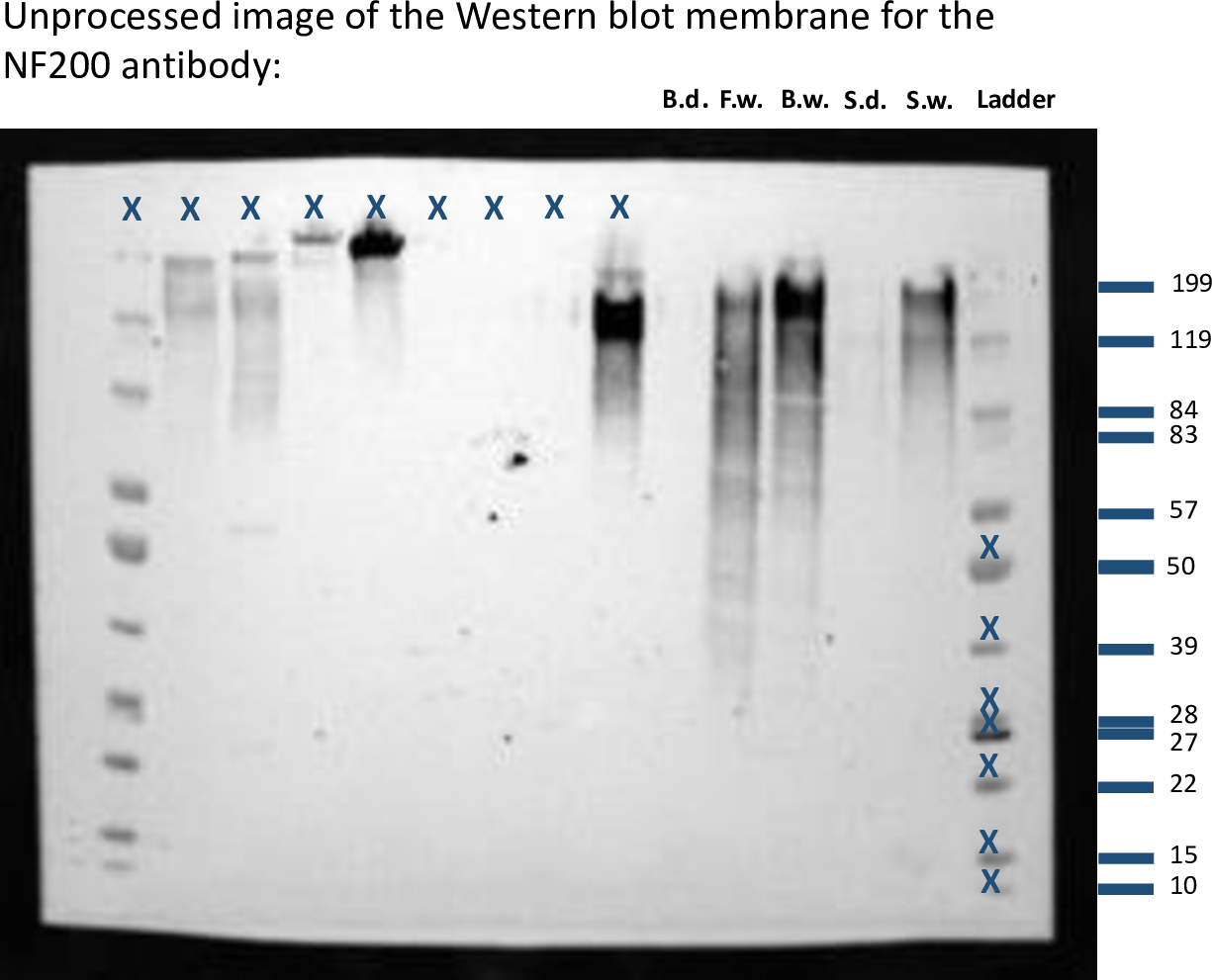

Supplement: S3 Fig — The blue “X” marks the lanes and rows that are not included in the final image. Nota bene: The 9 left lanes of these blots correspond to species not included in this study. (ZIP) [file pone.0269090.s003.zip › Supplementary_FigS3_4.tif]

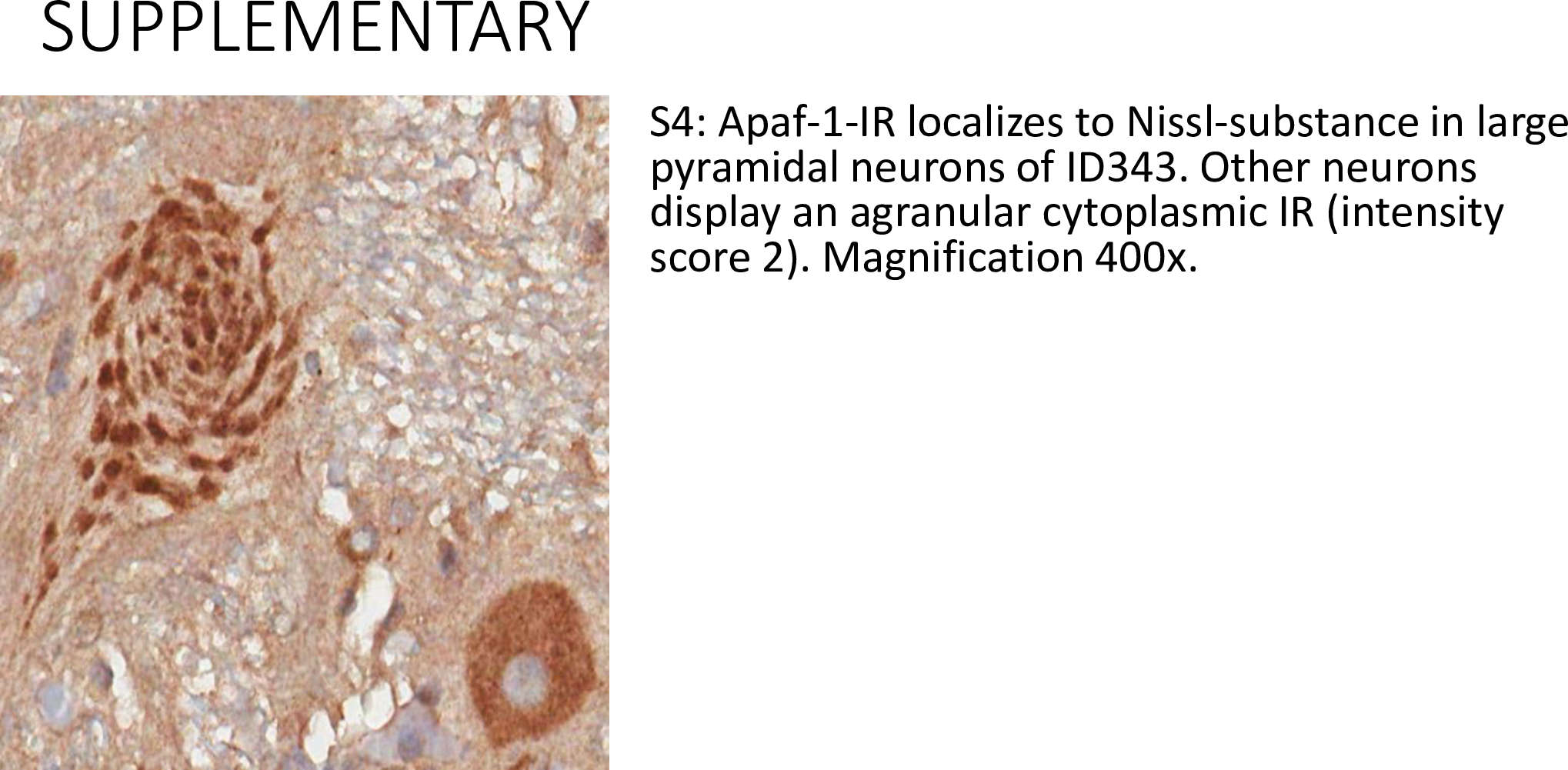

Supplement: S4 Fig — Other neurons display an agranularcytoplasmic IR (intensity score 2). Magnification 400x. (TIF) [file pone.0269090.s004.tif]

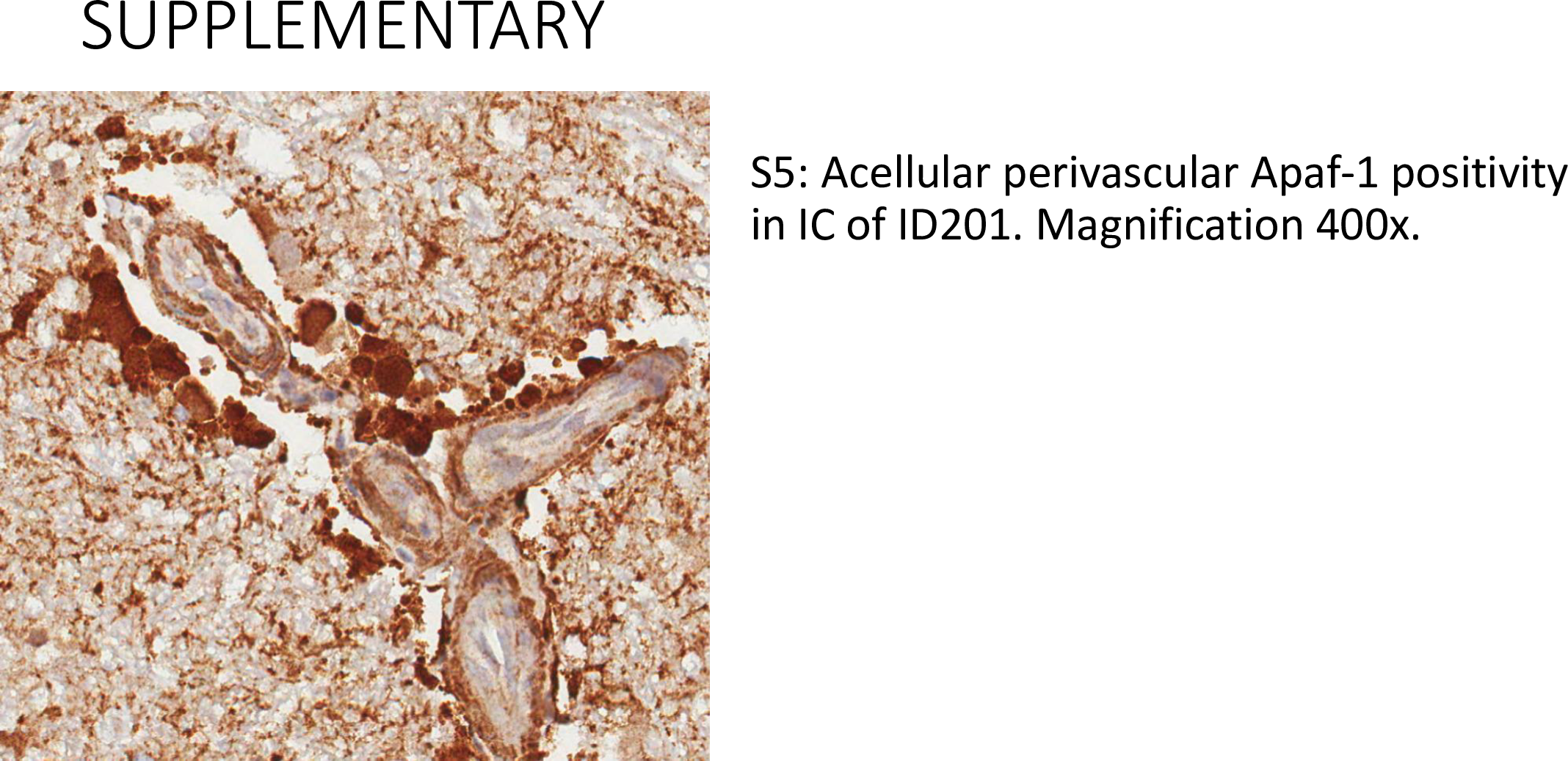

Supplement: S5 Fig — Magnification 400x. (TIF) [file pone.0269090.s005.tif]

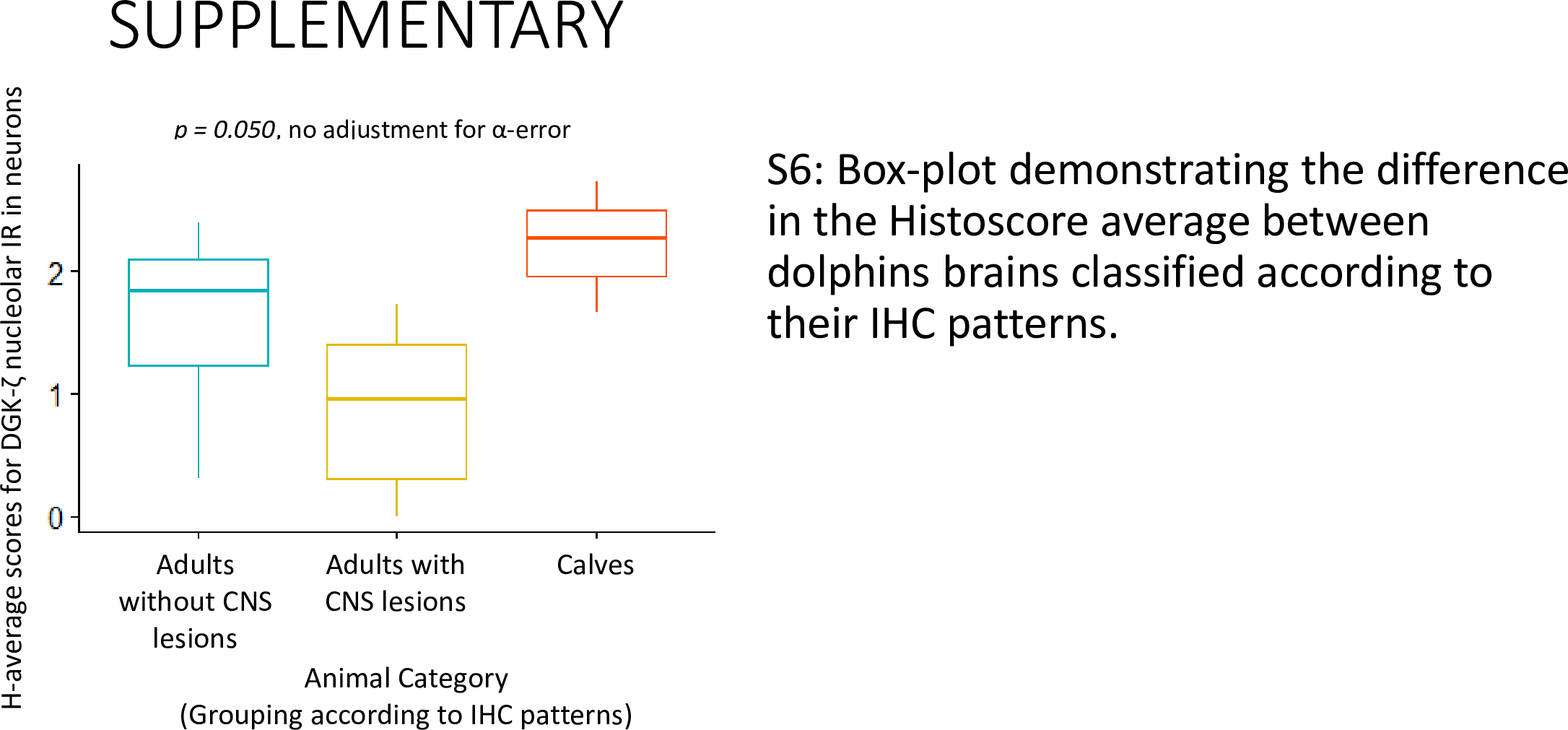

Supplement: S6 Fig — (TIF) [file pone.0269090.s006.tif]

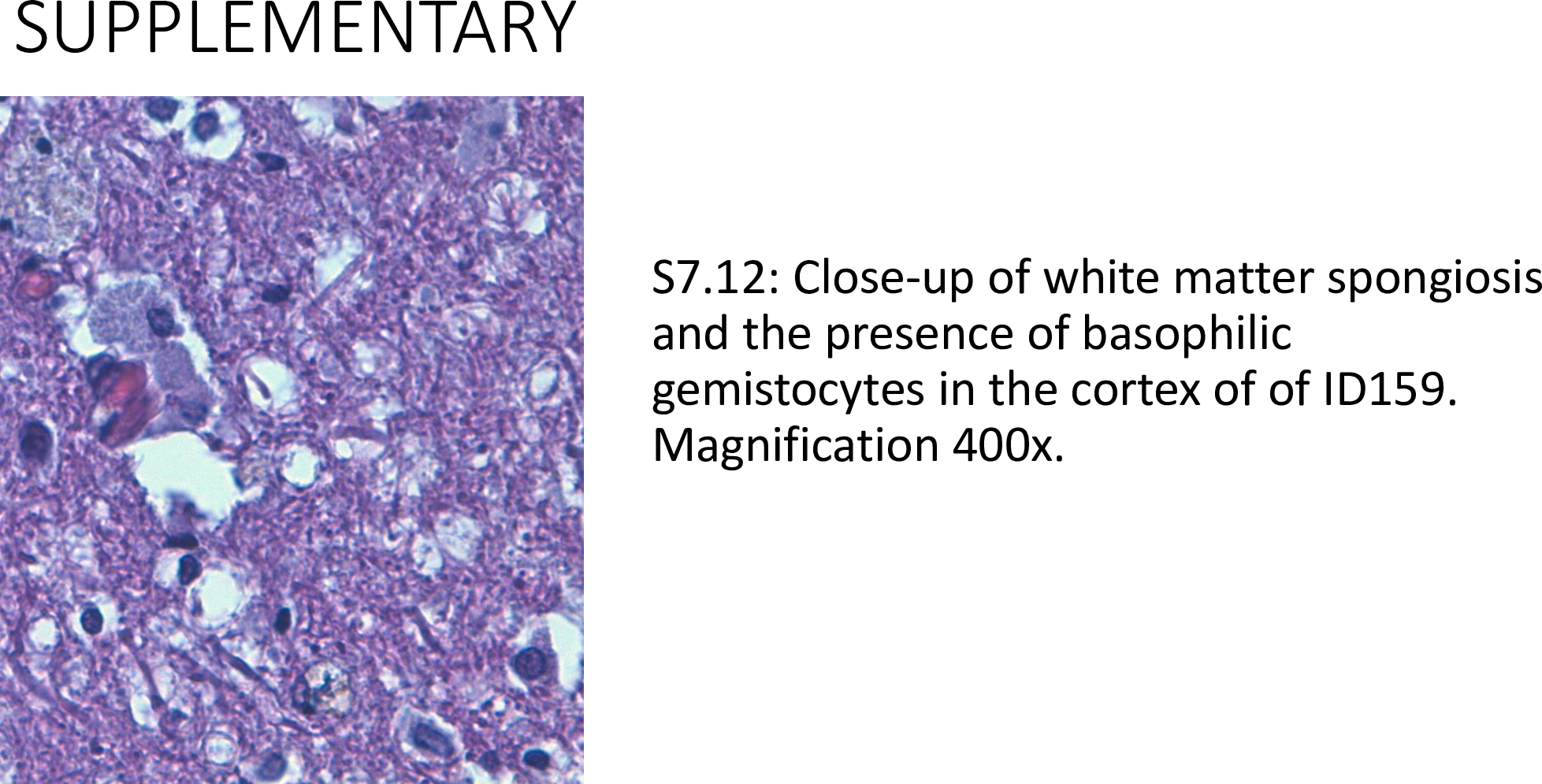

Supplement: S7 Fig — (ZIP) [file pone.0269090.s007.zip › Supplementary_FigS7_12.tif]

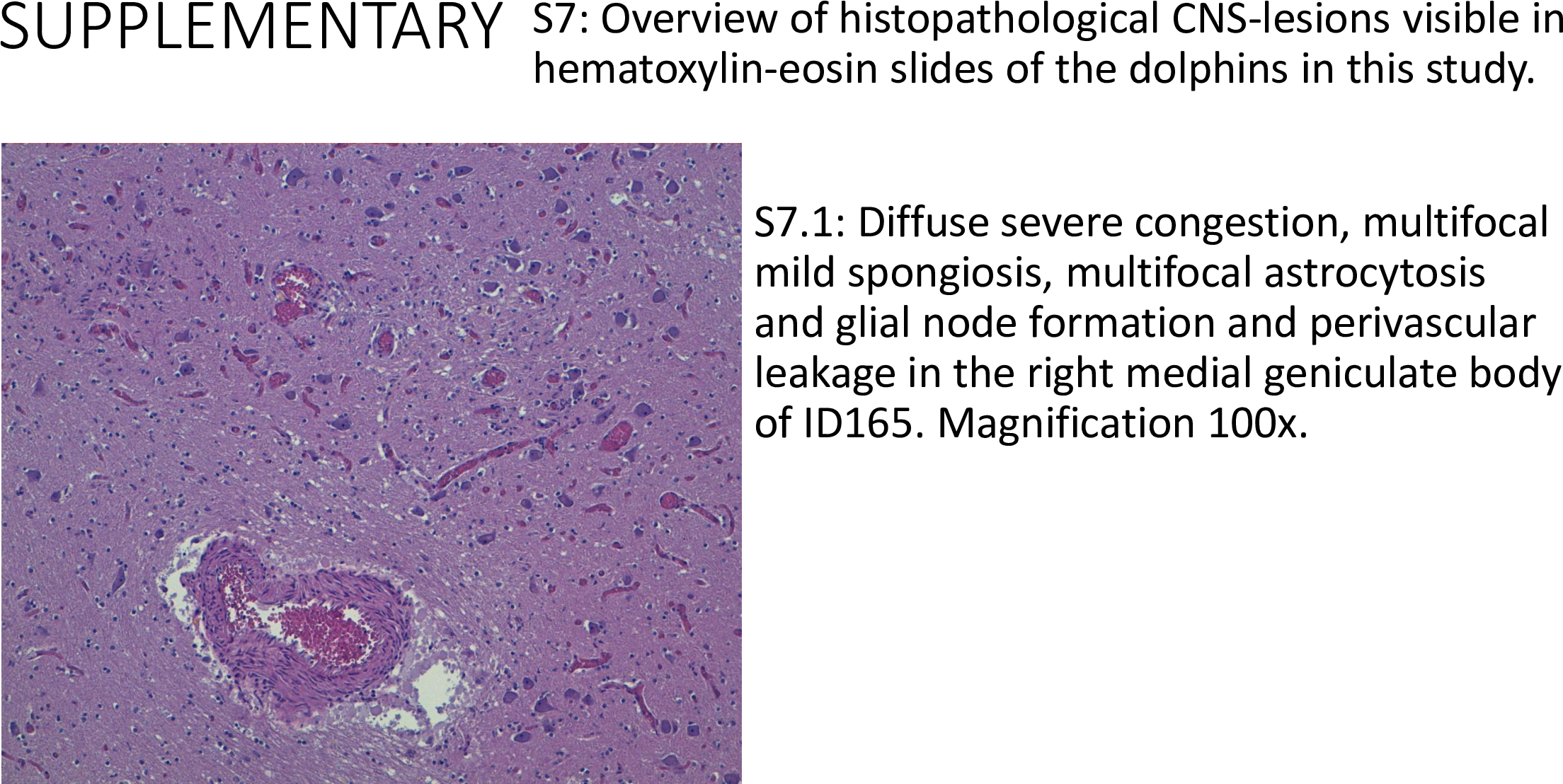

Supplement: S7 Fig — (ZIP) [file pone.0269090.s007.zip › Supplementary_FigS7_1.tif]

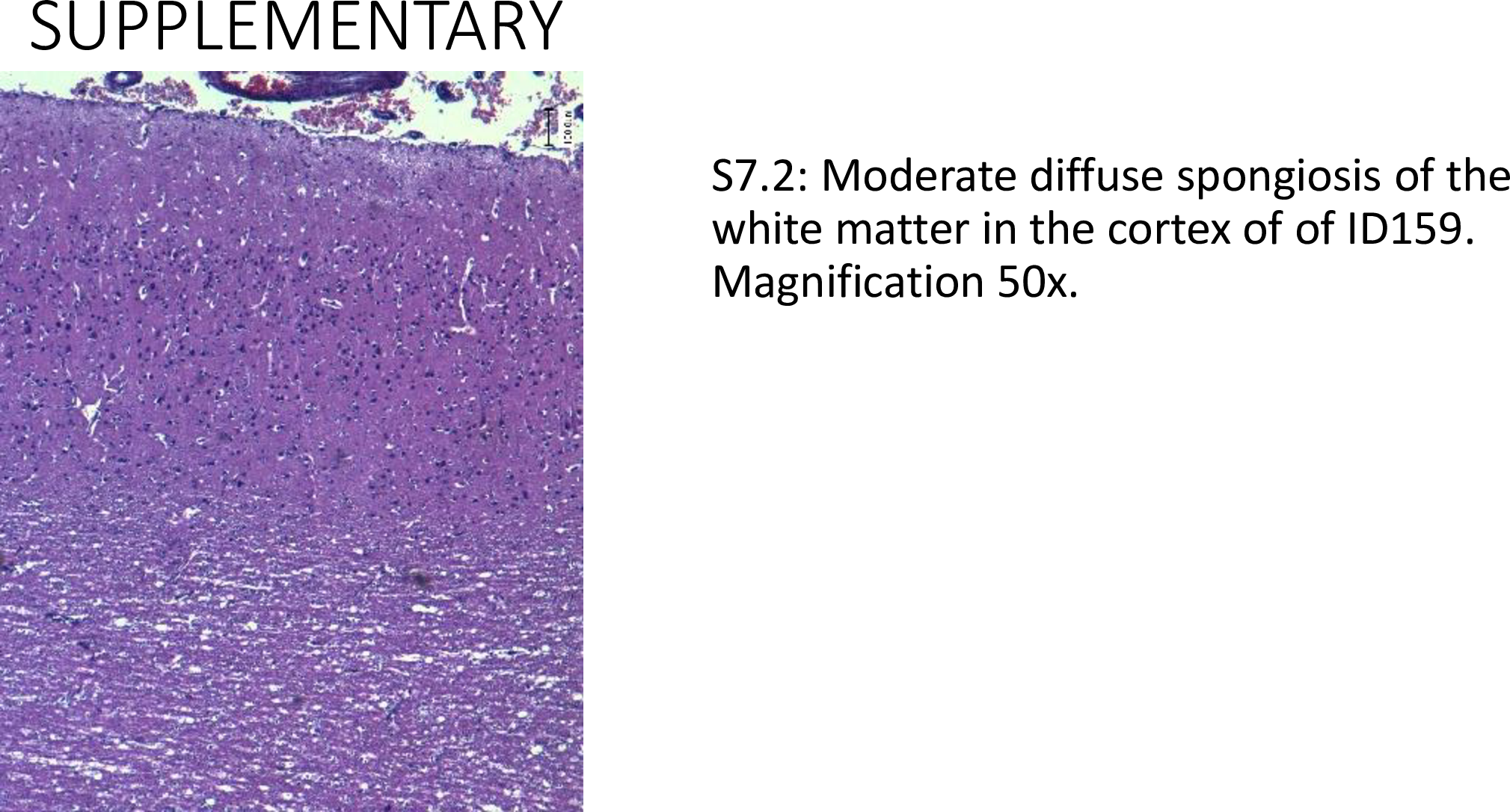

Supplement: S7 Fig — (ZIP) [file pone.0269090.s007.zip › Supplementary_FigS7_2.tif]

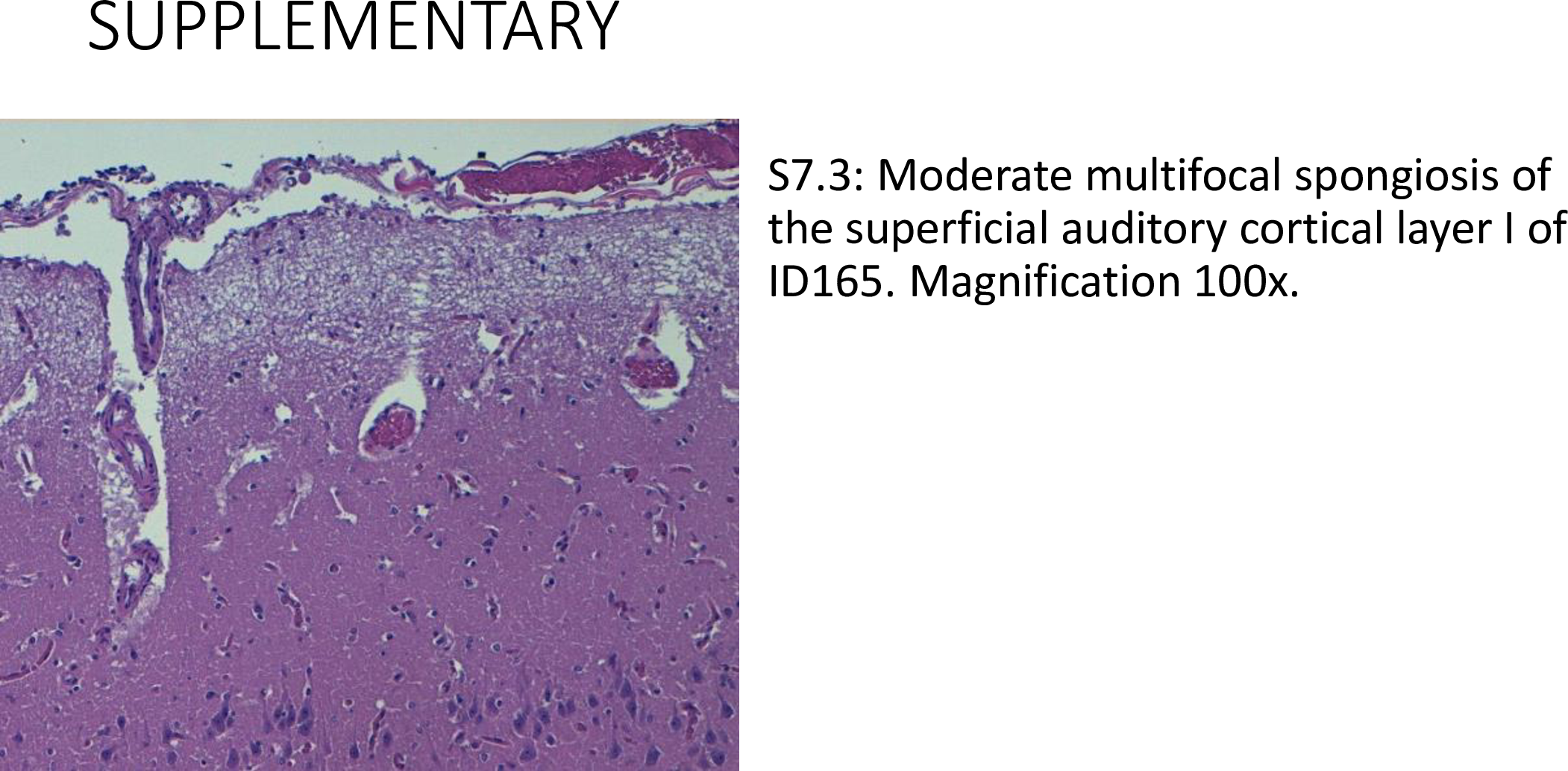

Supplement: S7 Fig — (ZIP) [file pone.0269090.s007.zip › Supplementary_FigS7_3.tif]

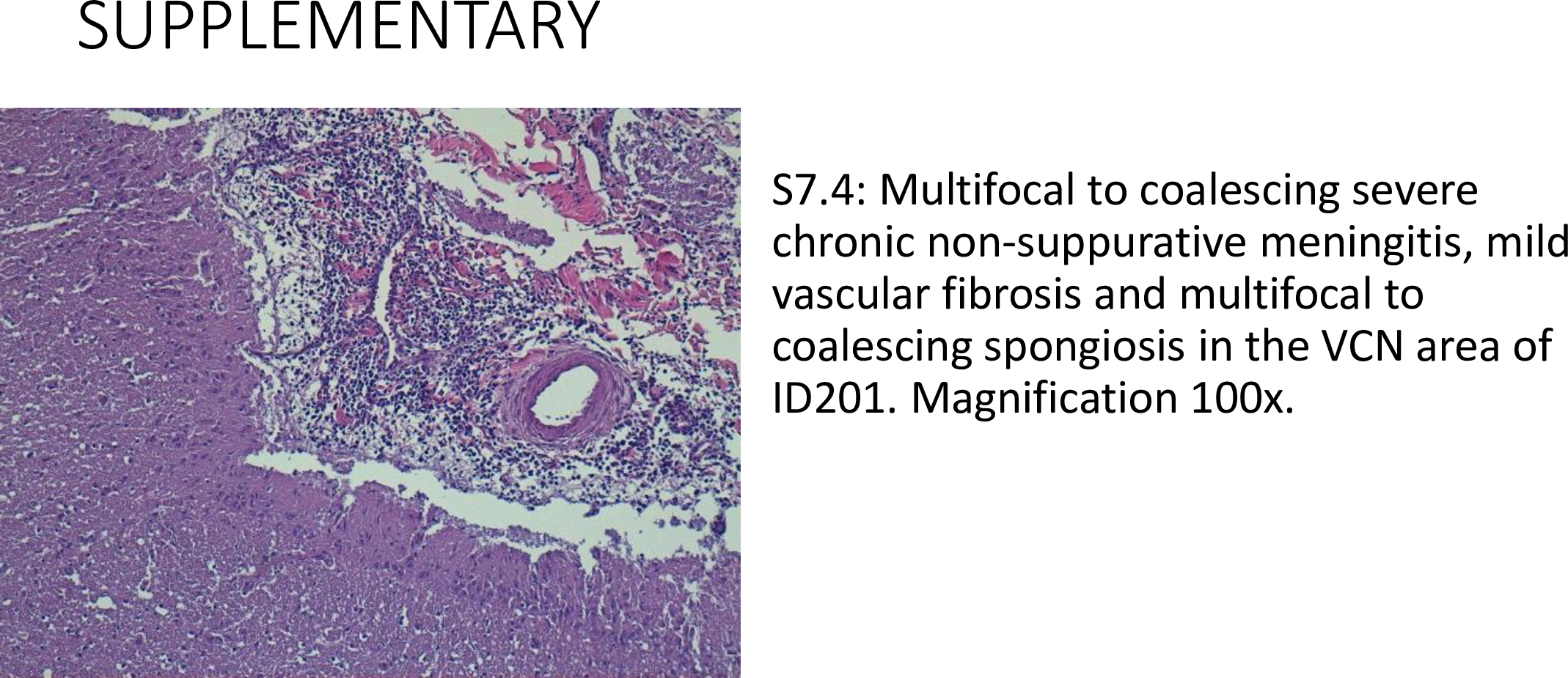

Supplement: S7 Fig — (ZIP) [file pone.0269090.s007.zip › Supplementary_FigS7_4.tif]

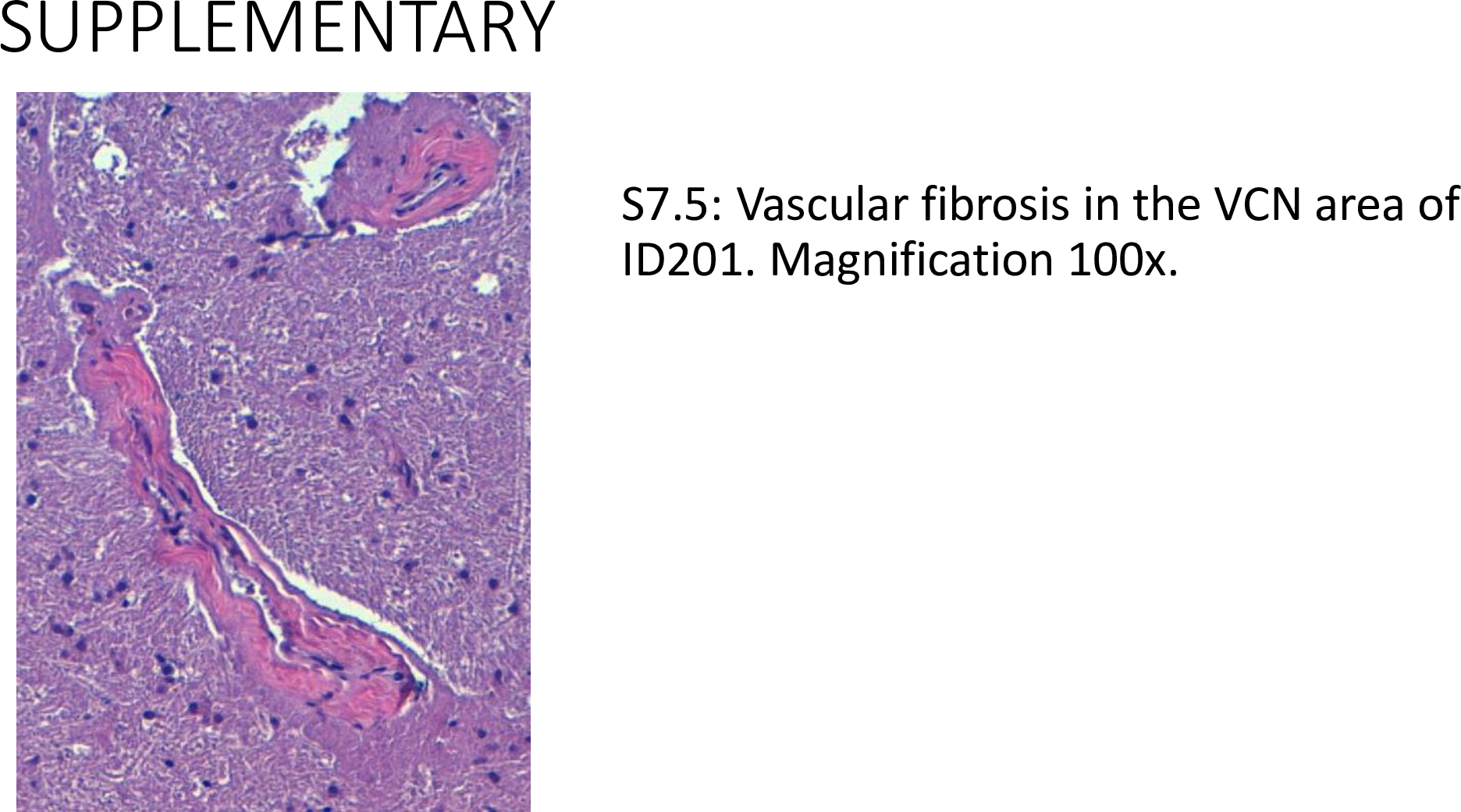

Supplement: S7 Fig — (ZIP) [file pone.0269090.s007.zip › Supplementary_FigS7_5.tif]

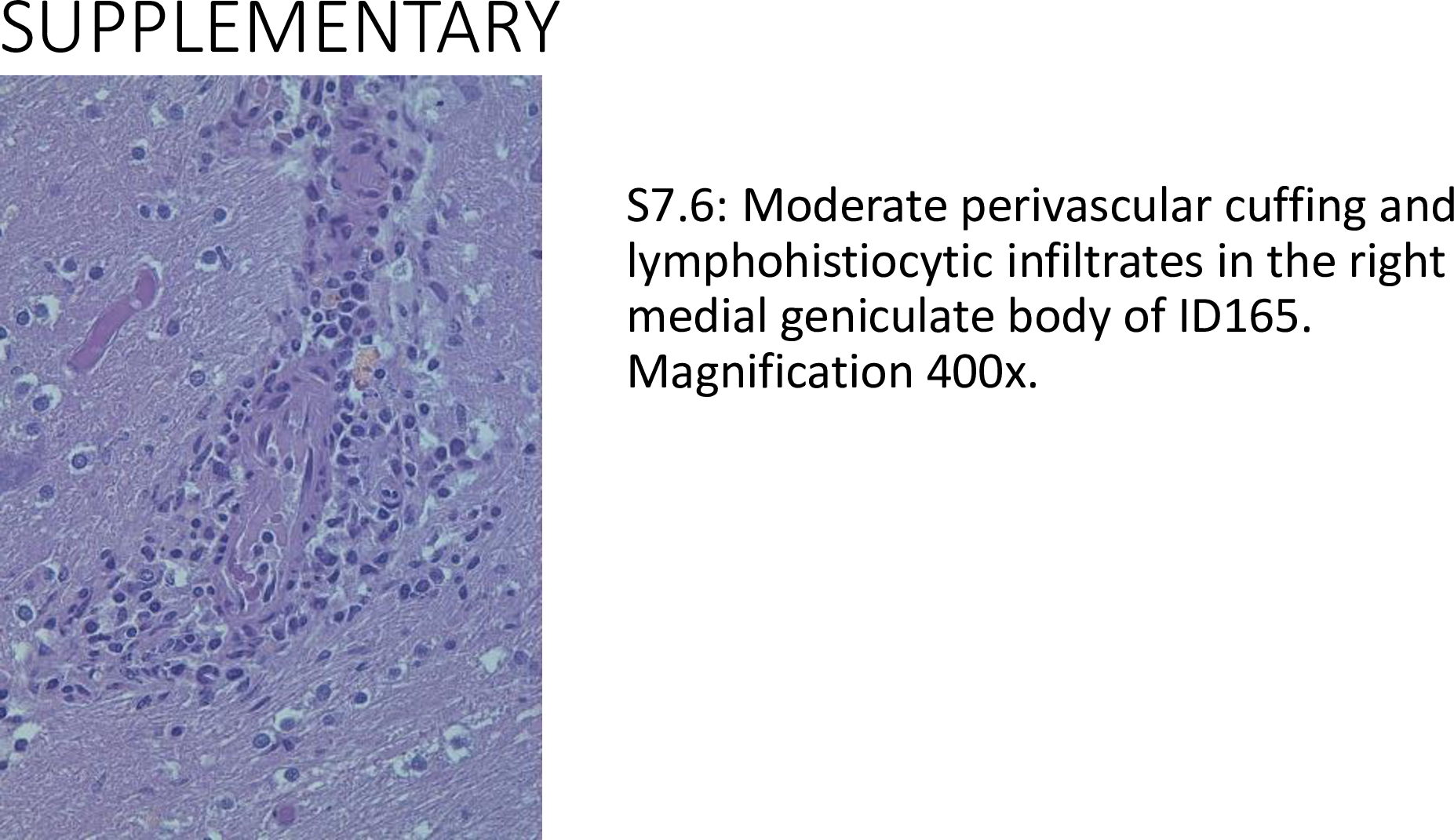

Supplement: S7 Fig — (ZIP) [file pone.0269090.s007.zip › Supplementary_FigS7_6.tif]

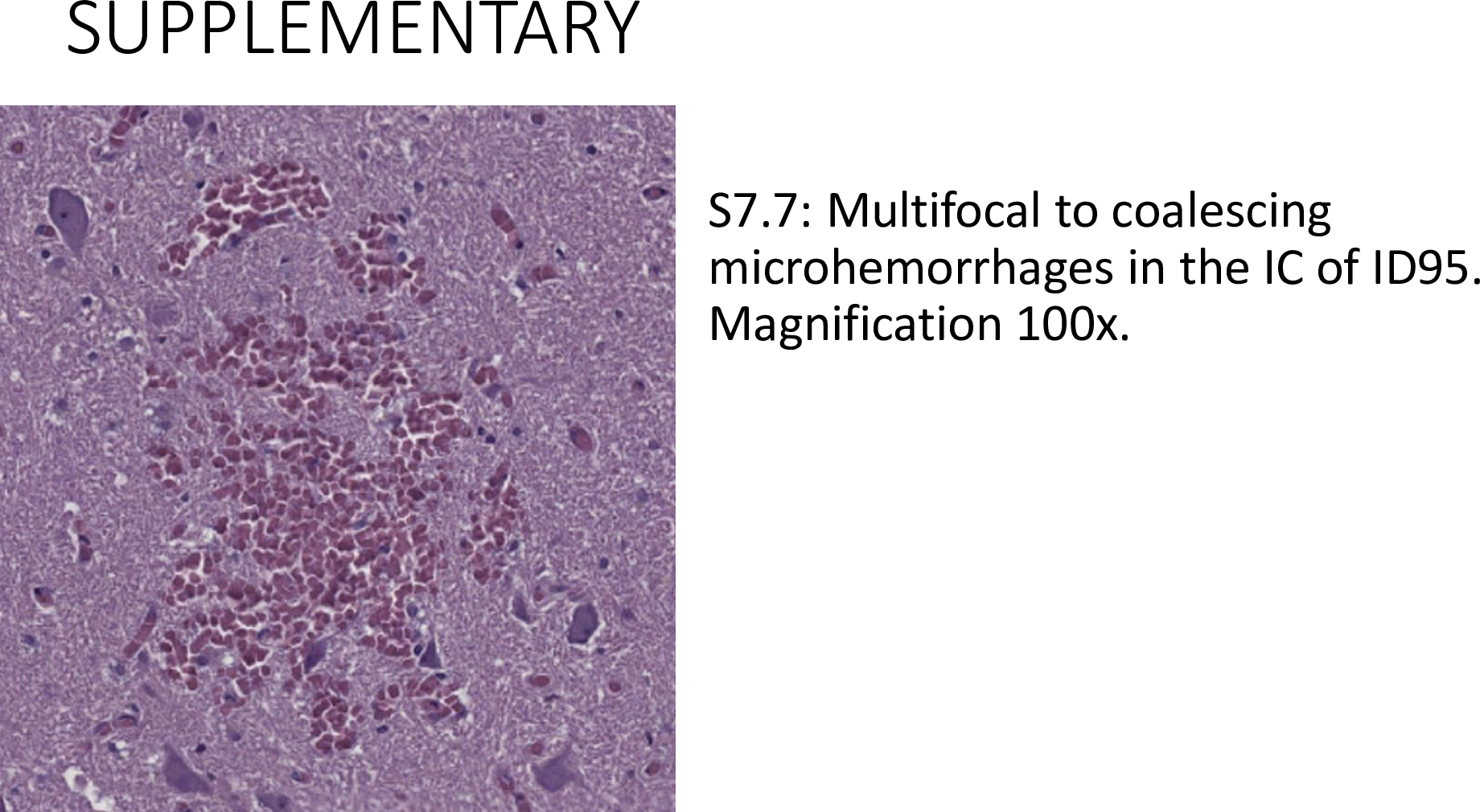

Supplement: S7 Fig — (ZIP) [file pone.0269090.s007.zip › Supplementary_FigS7_7.tif]

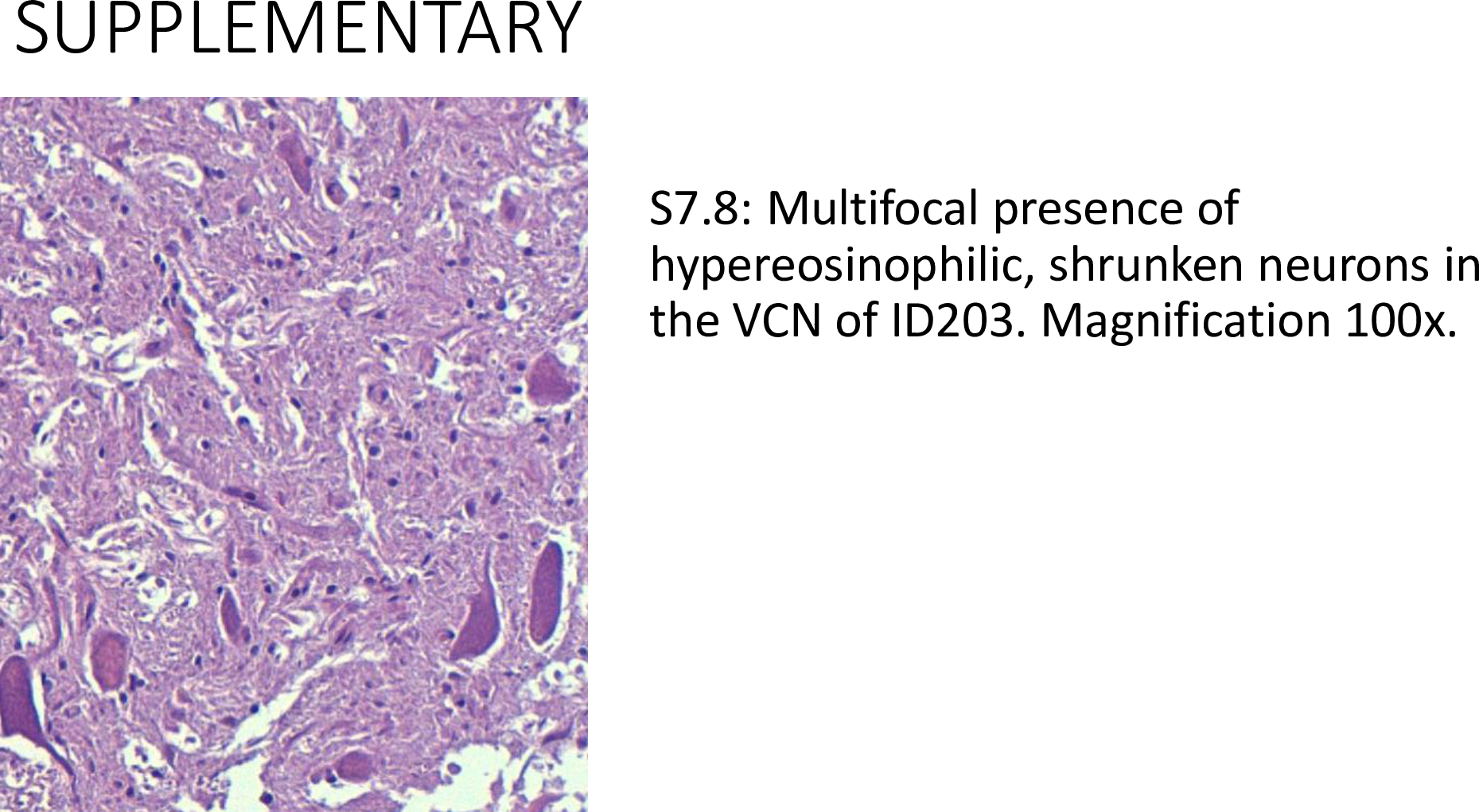

Supplement: S7 Fig — (ZIP) [file pone.0269090.s007.zip › Supplementary_FigS7_8.tif]

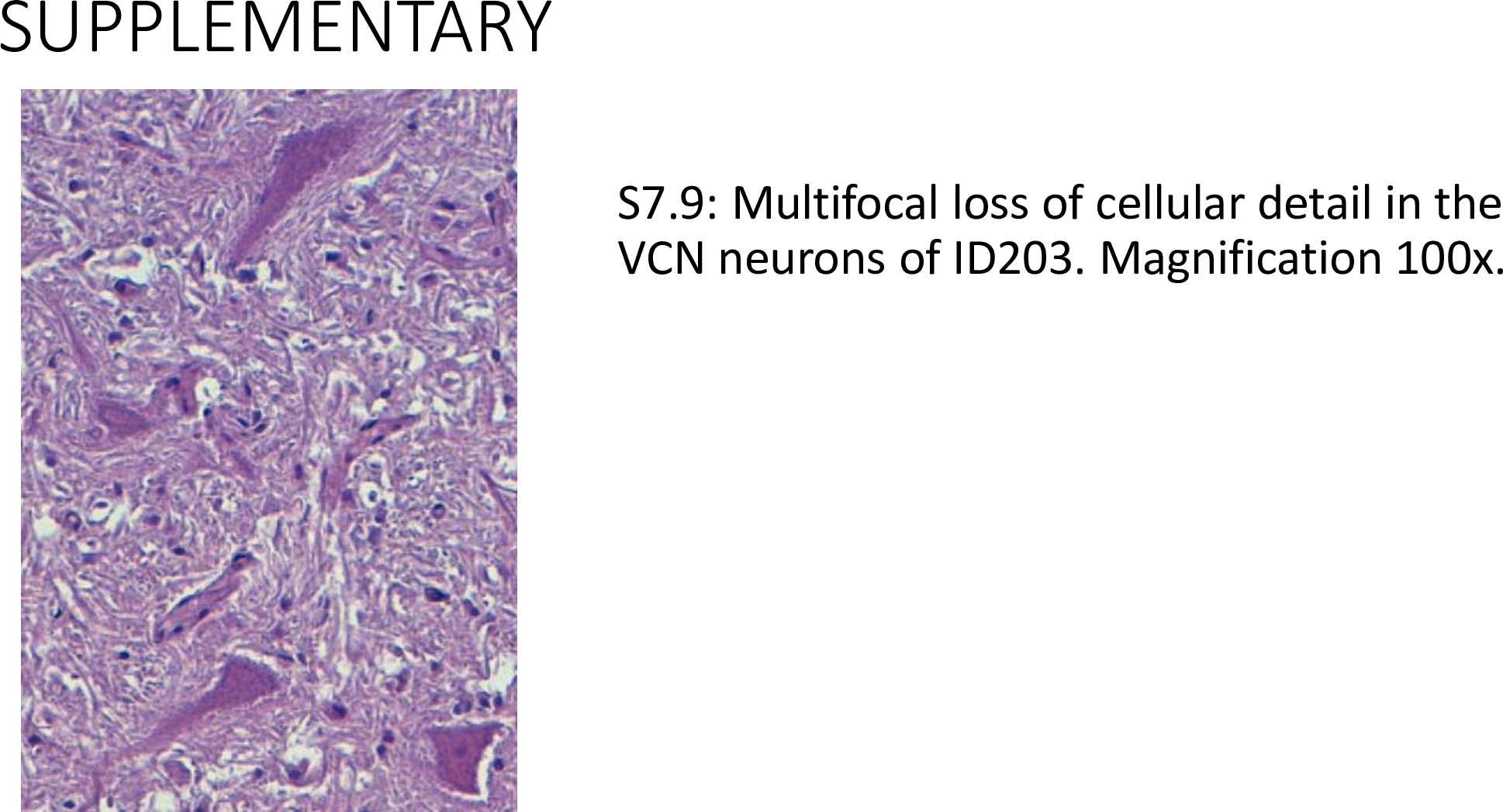

Supplement: S7 Fig — (ZIP) [file pone.0269090.s007.zip › Supplementary_FigS7_9.tif]

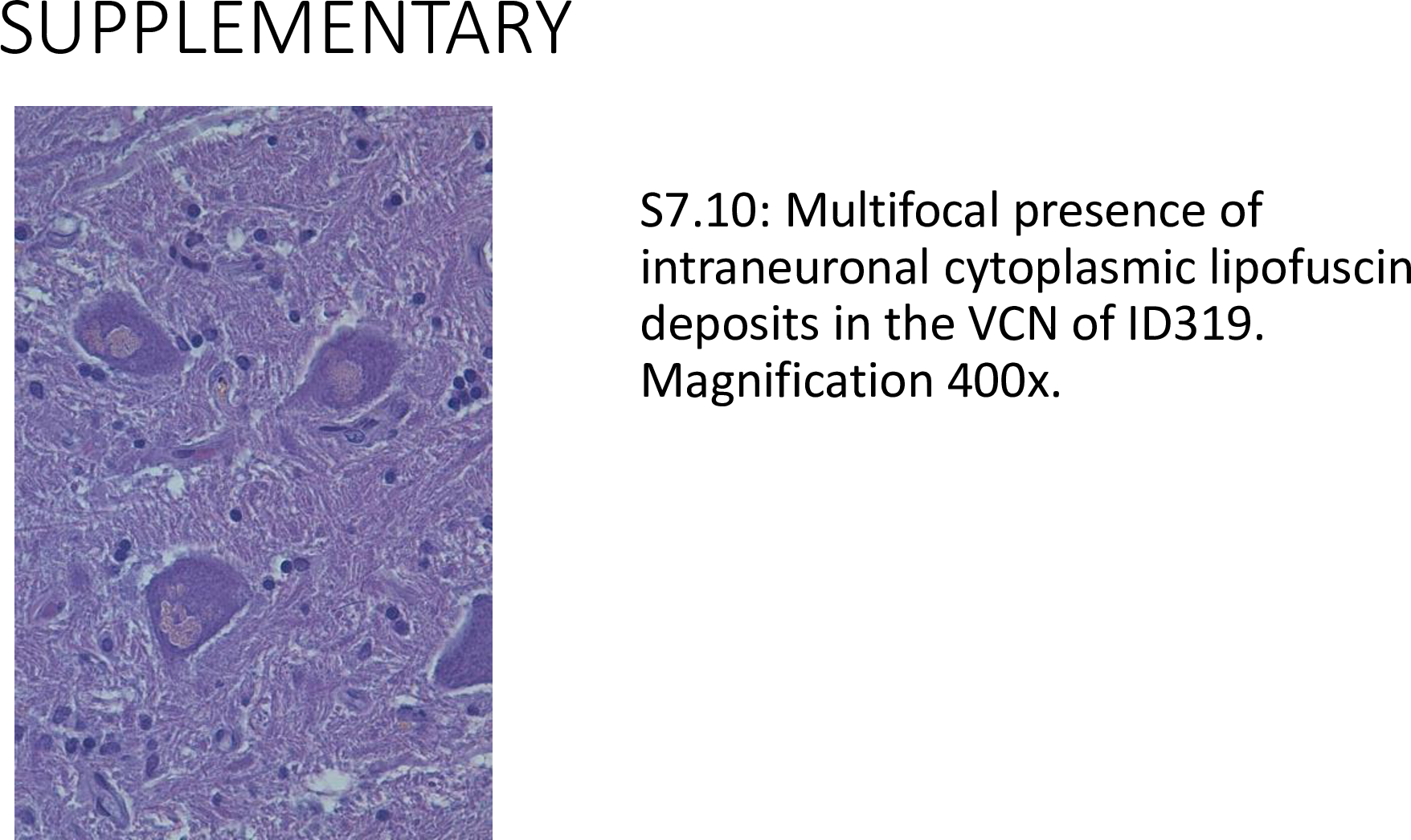

Supplement: S7 Fig — (ZIP) [file pone.0269090.s007.zip › Supplementary_FigS7_10.tif]

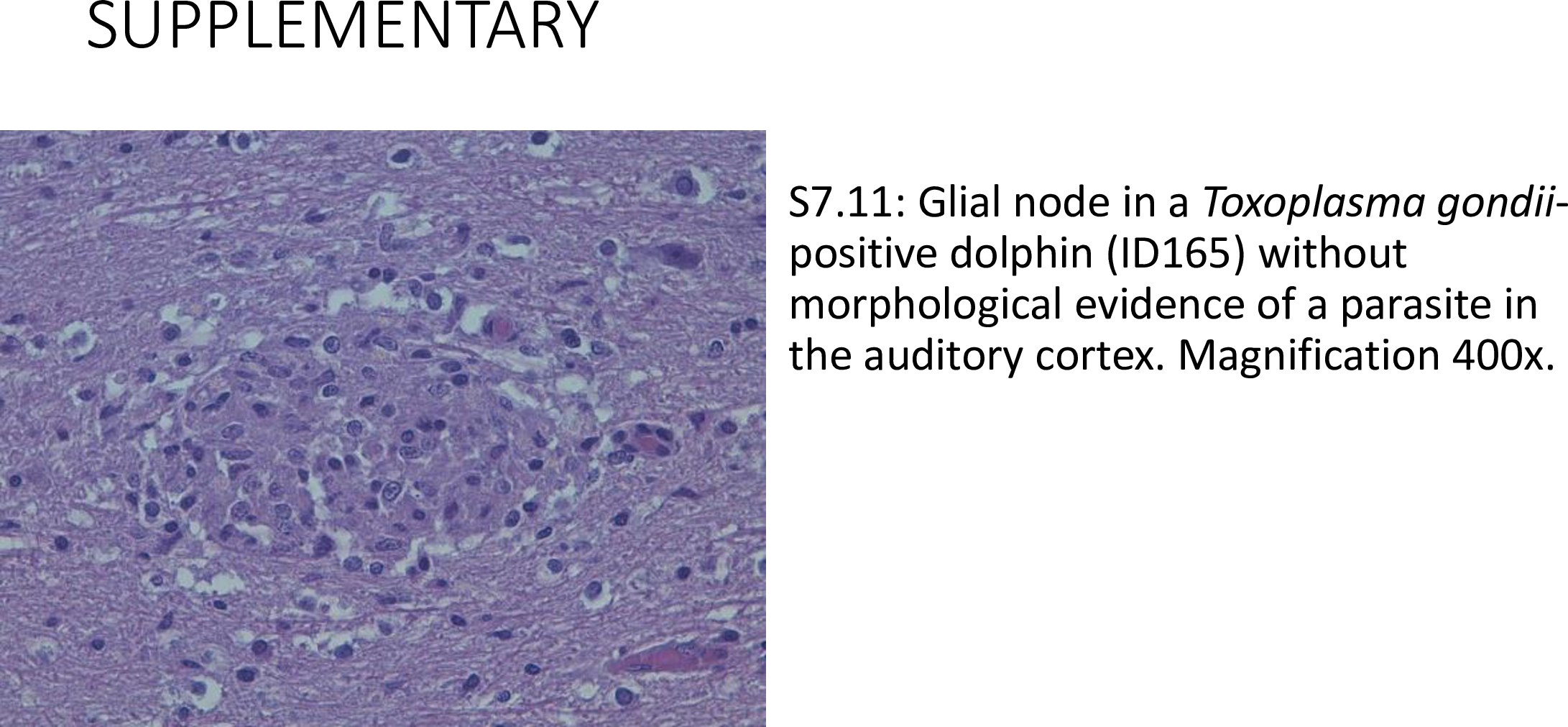

Supplement: S7 Fig — (ZIP) [file pone.0269090.s007.zip › Supplementary_FigS7_11.tif]
