# Supplementary material for: Systematic validation and assessment of immunohistochemical markers for central nervous system pathology in cetaceans, with emphasis on auditory pathways
Source: PLoS One. 2022 Jun 1;17(6):e0269090. doi: 10.1371/journal.pone.0269090 (PMC9159615; doi:10.1371/journal.pone.0269090)
Supplement: S1 Table — In gray—results that do not fit into the parametric/non-parametric category according to results of the Shapiro-Wilks and/or Levene’s tests. If the group differences persist in post-hoc analyses, level of adjustment for α-error are documented. Bf—Bonferroni adjustment for α-error. (PDF) [file pone.0269090.s008.pdf]

		Non-parametric	*p*	Parametric	*p*
A	IC	• Apaf1 astrocyte cytoplasmic H:U<br>• Apaf1 neuron cytoplasmic C:U/H<br>• Bcl2 neuron cytoplasmic C:U<br>Tendency:<br>• bAPP neuron nuclear<br>• NF200 neuron cytoskeletal	*0.026* Bf<br>Bf<br>none<br><br>*0.0628*<br>*0.0780*	• **Apaf1 astrocyte cytoplasmic H:U**<br>• Apaf1 neuron cytoplasmic C:U/H<br>• Bcl2 neuron cytoplasmic C:U<br>• bAPP neuron nuclear C:H	***0.0068***
	CN	• Apaf1 neuron cytoplasmic C:U<br>• bAPP neuron nuclear C:H<br>• bAPP astrocyte cytoplasmic C:U	Bf<br>Bf<br>Bf	• Apaf1 neuron cytoplasmic C:U<br>• Bcl2 neuron cytoplasmic C:H<br>• bAPP astrocyte cytoplasmic C:U	
B	IC	• **DGKz neuron nucleoli H:U**<br>• DGKz neuron nucleoli C:H/U<br>• Apaf1 neuron cytoplasmic C:U/H<br>• Bcl2 neuron cytoplasmic C:U<br>Tendency:<br>• **Apaf1 astrocyte cytoplasmic H:U**	None<br>Bf<br>Bf<br>Bf<br><br>None	• DGKz neuron nucleoli C:U<br>• **Apaf1 astrocyte cytoplasm H:U**<br>• Apaf1 neuron cytoplasm C:U/H<br>• bAPP neuron nuclear C:U/H<br>• Bcl2 neuron cytoplasmic C:U/H	 **0.0067**
	CN	• Apaf1 neuron cytoplasmic C:U/H<br>• bAPP neuron nuclear C:U/H<br>• bAPP astrocyte cytoplasmic C:U/H	Bf<br>Bf<br>Bf	• Apaf1 astrocyte cytoplasmic C:U/H<br>• Apaf1 neuron cytoplasmic C:U/H	
C	IC	• **DGKz neuron nucleoli Y:O**<br>• Apaf1 neuron cytoplasmic C:O>Y<br>• Bcl2 neuron cytoplasmic C:O<br>• bAPP astrocyte cytoplasmic C:O<br>• bAPP neuron nuclear C:O<br>Tendency:<br>• **DGKz neuron cytoplasmic Y:O**<br>• bAPP neuron nuclear Y:O	**0.0096 Bf**<br>Bf<br>Bf<br>Bf<br>None<br><br>0.0596<br>0.06 none	• **DGKz neuron nucleoli Y:O**<br>• DGKz neuron nucleoli C:O<br>• Apaf1 neuron cytoplasmic C:O>Y<br>• Bcl2 neuron cytoplasmic C:O>Y<br>• bAPP neuron nuclear C:O<br>• bAPP astrocyte cytoplasmic C:O	***0.0018***<br>0.002
	CN	• Apaf1 neuron cytoplasmic C:O<br>• bAPP neuron nuclear C:Y<br>• bAPP neuron nuclear C:Y>O<br>• bAPP astrocyte cytoplasmic C:Y<br>Tendency:<br>• DGKz neuron nucleoli<br>• Apaf1 neuron cytoplasmic C:Y<br>• Bcl2 neuron cytoplasmic	None<br>Bf<br>None<br>Bf<br><br>0.0751<br><br>0.0694	• Apaf1 neuron cytoplasmic C:O<br>• Bcl2 neuron cytoplasmic C:Y<br>• bAPP neuron nuclear C:Y<br>• bAPP astrocyte cytoplasmic C:Y>O<br>Tendency:<br>• **DGKz neuron nucleoli**	 <br><br><br><br>**0.0786**

Supplementary Table 1: Results of the statistical comparisons of groups made according to A) morphopathological findings in haematoxylin-eosin stained sections, B) immunohistochemical findings C) age. In gray—results that do not fit into the parametric/non-parametric category according to results of the Shapiro-Wilks and/or Levene's tests. If the group differences persist in post-hoc analyses, level of adjustment for α-error are documented. Bf—Bonferroni adjustment for α-error.
