# Supplementary material for: Systematic validation and assessment of immunohistochemical markers for central nervous system pathology in cetaceans, with emphasis on auditory pathways
Source: PLoS One. 2022 Jun 1;17(6):e0269090. doi: 10.1371/journal.pone.0269090 (PMC9159615; doi:10.1371/journal.pone.0269090)
Supplement: S2 Table — (PDF) [file pone.0269090.s009.pdf]

ID	Sex	Body length (cm)	Full histopathological findings in haematoxylin-eosin stained central nervous system tissues	Probable cause of death
**146**	M	226	Mild neuronal degeneration and necrosis, perivascular edema and congestion	Hypovolemic shock
**20**	F	NA	Mild congestion, perivascular edema and superficial cortical spongiosis	Could not be determined
**89**	M	NA	Mild neuronal degeneration and necrosis. Mild diffuse spongiosis	Shock and metabolic acidosis
**139**	M	268	Mild neuronal degeneration and necrosis	Hypovolemic shock and hepatic necrosis
**159**	M	328	Mild multifocal spongiosis in white matter (WM) and superficial cortex, mild vasculitis, astrocytosis and satellitosis. Multifocal presence of gemistocytes in WM, multifocal neuronal necrosis. Multifocal presence of lipofuscin in Purkinje neurons	Septic shock
**319**	M	310	Multifocal lymphohistiocytic spinal cord pachymeningitis, with presence of corpora amylacea. Presence of lipofuscin in neurons and spongiosis of the WM and superficial cortical layers. Intravascular neutrophil margination and clotting. Focal evidence of few shrunken, hypereosinophilic pyramidal neurons	Septic shock
**344**	M	195	Diffuse, moderate-to-severe congestion	Could not be determined
**192**	F	240	No abnormality detected	Could not be determined
**196**	M	300	Moderate chronic multifocal meningoencephalomyelitis with associated multifocal demyelination and non-suppurative vasculitis. Multifocal presence of glial nodes with isolated *T. gondii* cysts	Hepatic insufficiency, parasitic encephalitis and enteritis
**95**	F	285	Severe multifocal vasogenic edema, moderate astrocytosis and congestion. Mild perivascular cuffing and multifocal coalescing neuronal necrosis. Multifocal microhemorrhages in IC	Stranding pathology, with a concomitant severe, cholesteatoma-like lesion on the left occipital cortex
**107**	M	250	Multifocally few necrotic cerebellar Purkinje neurons. Mild diffuse cerebral spongiosis. Mild satellitosis and multifocal perivascular and meningeal lymphocytic infiltration, with presence of glial nodes	Respiratory insufficiency due to severe pulmonary edema and mycotic pneumonia. Moderate diffuse edema and multifocal mild vasculitis in the brain.
**133**	F	248	Mild diffuse spongiosis and congestion, mild focal perivascular cuffing and astrocytosis. Mild multifocal neuronal degeneration in cortex. Multifocal necrosis of Purkinje neurons	Cardiovascular shock, necrotic enteritis
**142**	F	256	Multifocal necrotic neurons with moderate satellitosis, astrocytosis, gliosis and perivascular cuffing. Multifocal presence of glial nodes with occasional *T. gondii* cysts at the center	Septic shock following systemic mycosis and cerebral toxoplasmosis
**165**	M	280	Multifocal necrotic neurons with moderate satellitosis, astrocytosis and perivascular cuffing. Multifocal presence of glial nodes with occasional *T. gondii* cysts at the center	Septic shock following systemic and cerebral toxoplasmosis
**201**	M	297	Multifocal to coalescing severe chronic non-suppurative meningitis and moderate to severe vasculitis with lymphohistiocytic infiltrates, vascular fibrosis, multifocal foci of mild to moderate astrocytosis, neuronal necrosis and satellitosis. Focal cortical presence of protozoan parasitic cyst. Moderate congestion.	Septic shock,<br>severe monolateral pneumonia, morbilliviral infection
**203**	M	284	Mild focal satellitosis, mild congestion, and loss of cellular detail	Cardiovascular shock
**114**	M	NA	Mild multifocal astrocytosis and astrogliosis, with mild-to-moderate diffuse congestion. Mild multifocal neuronal necrosis	Meconium aspiration syndrome
**144**	M	NA	Mild multifocal neuronal necrosis	Meconium aspiration syndrome
**145**	M	118	Mild multifocal neuronal necrosis	Meconium aspiration syndrome
**343**	F	95.5	Mild multifocal neuronal necrosis	Meconium aspiration syndrome

Supplementary Table S2: Summary of the ID, sex, body length, full histopathological findings of the CNS, and the most probable cause of death of the dolphins included in this study. **C**—cal. **M**—male; **F**—female.
